# Editing of mouse and human immunoglobulin genes by CRISPR-Cas9 system

**DOI:** 10.1038/ncomms10934

**Published:** 2016-03-09

**Authors:** Taek-Chin Cheong, Mara Compagno, Roberto Chiarle

**Affiliations:** 1 Department of Pathology, Children's Hospital Boston and Harvard Medical School, Boston, Massachusetts 02115, USA; 2 Department of Molecular Biotechnology and Health Sciences, University of Torino, Torino 10126, Italy

## Abstract

Applications of the CRISPR-Cas9 system to edit the genome have widely expanded to include DNA gene knock-out, deletions, chromosomal rearrangements, RNA editing and genome-wide screenings. Here we show the application of CRISPR-Cas9 technology to edit the mouse and human immunoglobulin (Ig) genes. By delivering Cas9 and guide-RNA (gRNA) with retro- or lenti-virus to IgM^+^ mouse B cells and hybridomas, we induce class-switch recombination (CSR) of the IgH chain to the desired subclass. Similarly, we induce CSR in all human B cell lines tested with high efficiency to targeted IgH subclass. Finally, we engineer mouse hybridomas to secrete Fab′ fragments instead of the whole Ig. Our results indicate that Ig genes in mouse and human cells can be edited to obtain any desired IgH switching helpful to study the biology of normal and lymphoma B cells. We also propose applications that could transform the technology of antibody production.

## Abstract (web summary)

CRISPR-Cas9 has been used to generate a range of genetic modifications including gene knock-outs and chromosomal rearrangements. Here the authors target the immunogloblin genes and demonstrate the induction of class switch recombination, opening up possibilities for research and antibody production.

## Graphical abstract

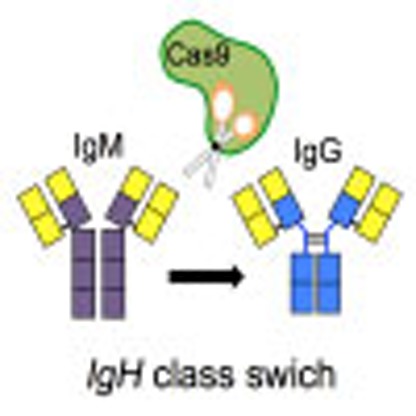

Gene rearrangements editing the immunoglobulin (Ig) genes such as V(D)J recombination and class-switch recombination (CSR) require the formation of DNA double-strand breaks (DSBs) as the key initiating step^1^,^2^,^3^. In physiological conditions, DSBs are introduced at the Ig genes by the activity of B-cell-specific enzymes such as recombination-activating gene 1/2 (RAG1/2) and activation-induced cytidine deaminase (AID)^1^,^2^,^3^. During CSR, AID generates DSBs in the Ig locus by targeting repetitive sequences in the switch (S) regions that precede each Ig heavy (IgH) coding sequence^1^,^2^,^3^. Paired DSBs in the switch regions are then joined by the classical and alternative non-homologous end-joining (NHEJ) pathways to generate a switch of the IgH^4^. This long range joining is thought to be part of a general mechanism of DNA repair where two DSBs are joined in *cis* over long chromosome distances^5^. Indeed, efficient CSR can be obtained in absence of AID or S regions after the introduction of DSBs by site-specific I-SceI endonuclease^6^.

The bacterial type II clustered regularly interspaced short palindromic repeat (CRISPR)-Cas9 (CRISPR-associated Protein 9) systems have great potentials for RNA-guided genome editing, including multiplexing genome engineering, gene targeting by homologous recombination, regulation of transcription, chromosomal translocation formation, high-throughput functional genomic screens and even RNA editing^7^,^8^,^9^,^10^. We and others demonstrated that when two DSBs are simultaneously introduced in a cell *in vitro* or *in vivo* by CRISPR-Cas9 activity, a variety of gene rearrangements are generated, including large deletions (up to 12 Mb), inversions and chromosomal translocations^11^,^12^,^13^,^14^.

Here we show that the CRISPR-Cas9 system can be adapted to efficiently induce CSR in primary mouse B cells and human B-cell lines. In addition, this system can be proficiently used to engineer hybridoma cells to produce monoclonal antibody with a switched Ig heavy chain or to secrete the immunoglobulin Fab′ fragments only.

## Results

### CRISPR-Cas9-mediated CSR in mouse B cells

**Figure 1:**
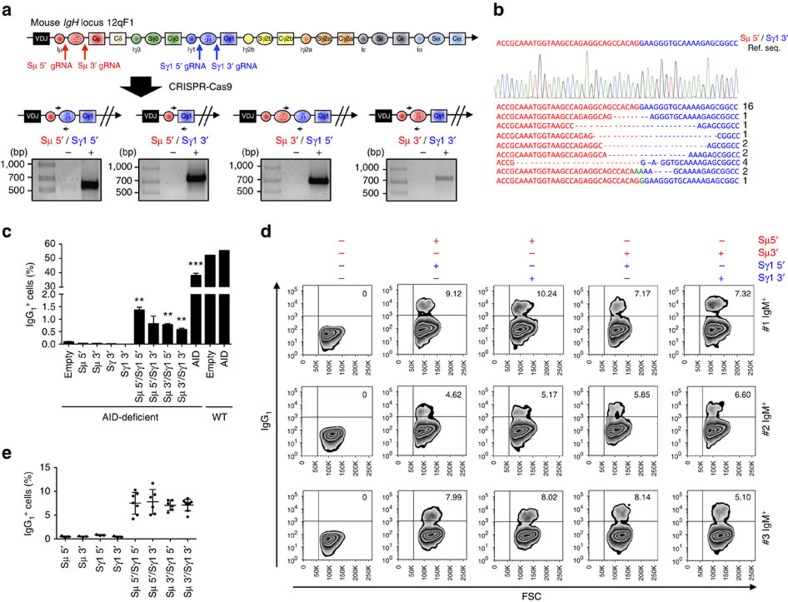
Induction of class-switch recombination (CSR) by CRISPR-Cas9 system in mouse cells. (**a**) Top: genomic organization of the mouse *IgH* constant region locus and position of the gRNAs used in this study. Bottom: schematic representation of four possible CSR products induced by deletion of DNA segments between Sμ and Sγ1 regions. Black arrows indicate the PCR primers designed to sequence the deletions. Gels show PCR amplicons obtained with the indicated primers. (**b**) An example chromatogram showing a perfect Sμ 5′ and Sγ1 3′ genomic junction, as well as representative sequences of junctions identified from 30 clones. Ref. Seq., sequence of the predicted genomic junction between Sμ 5′ and Sγ1 3′ regions. Green: insertions; Dashes: deleted bases. (**c**) Mouse B cells isolated from the spleen of 129S2 WT and AID-deficient mice were activated by anti-CD40 antibody and IL-4 for 1 day and then transduced with retrovirus expressing Cas9 nuclease and gRNAs used in (**a**). Empty GFP- or AID- expressing retroviruses were used as negative or positive controls, respectively. At day 4, cells were collected, stained with IgG_1_ antibody, and then analysed by flow cytometry. IgG_1_^+^ cells were gated on GFP-positive population. Mean±s.d.; *n*=3 biological replicates for each condition. Statistical analysis determined using unpaired *t*-test (**P<0.01; ***P<0.001). (**d**,**e**) IgM^+^ hybridomas were transduced with four different combinations of lentiviruses expressing Cas9 nuclease and gRNAs as above. Representative zebra plots (**d**) and average percentages±s.d. of CSR (**e**) from six independent experiments are presented.

Since CSR is a DNA deletion induced by two DSBs occurring in the S regions preceding the IgH constant sequences, we sought to engineer CSR by CRISPR-Cas9-mediated DNA deletion. We first designed a system to target the mouse *IgH* locus. Given that S regions are highly repetitive, we generated lentiviral vectors expressing Cas9 and guide-RNA (gRNA) targeting the more specific DNA sequences flanking immediately upstream (Sμ 5′ gRNA and Sγ1 5′ gRNA) or downstream (Sμ 3′ gRNA and Sγ1 3′ gRNA) of the S regions that precede the mouse Cμ and Cγ1 IgH constant sequences (Fig. 1a and Supplementary Fig. 1a). Efficiency of selected gRNA sequences was tested by Surveyor assay (Supplementary Fig. 1b). By introducing simultaneous DSBs in sites flanking the Sμ and Sγ1 regions, the prediction was to generate deletions of the DNA segment encompassed by the two DSBs as well as excision circles in a process closely mimicking CSR occurring in B cells^3^. To test this prediction, we first transduced immortalized mouse fibroblasts. PCRs with specific primers confirmed that the expected deletions were obtained with all four gRNA combinations (Fig. 1a). Sanger sequencing of PCR products demonstrated not only the expected DNA junctions with a predominance of precise junctions between the Cas9-mediated DSBs, but also 5′ or 3′ deletions and insertions, as previously described^11^,^12^,^13^,^14^ (Fig. 1b). PCR also detected the expected excision circles as well as inversions of the DNA segment encompassed by the DSBs, as we previously demonstrated in a different model^13^ (Supplementary Fig. 2a,b).

Next we sought to induce CSR in primary mouse B cells. When activated *in vitro* by anti-CD40 antibody and Interleukin-4 (IL-4), mouse B cells typically are induced to high levels of CSR (Supplementary Fig. 3c)^15^, impairing a precise assessment of CSR induced by the CRISPR-Cas9 system. Thus, we decided to exploit AID-deficient B cells in which CSR is practically undetectable^15^. Since retroviruses are more efficient to transduce primary B cells than lentiviruses, we generated a retroviral vector that expressed Cas9 and the same gRNAs used in fibroblasts (Supplementary Fig. 3a,b). Remarkably, with all four Cas9–gRNA combinations, we observed a small but distinct population of AID-deficient B cells that switched from IgM to IgG_1_ (Fig. 1c; Supplementary Fig. 3d). The overall frequency of 1% CSR was in line with previous reports describing chromosomal translocations induced by CRISPR-Cas9 (refs 11, 12, 13, 14).

### CRISPR-Cas9-mediated CSR in mouse hybridoma cells

To further validate the efficiency of the CRISPR-Cas9 system to induce CSR in mouse cells, we transduced IgM^+^ hybridoma cells. Surprisingly, higher levels of CSR than in primary B cells were observed in all hybridomas tested with all gRNA combinations (range 4–11%; Fig. 1d,e). Remarkably, similar levels of CSR were observed when the entire Sμ and Sγ1 regions were conserved (Sμ 3′ gRNA with Sγ1 5′ gRNA) or deleted (Sμ 5′ gRNA with Sγ1 3′ gRNA; Fig. 1a,d,e). By single-cell cloning, we isolated pure IgG_1_ hybridomas at the expected frequency (12 pure IgG_1_ clones out of 189 total clones: 6.3%; Supplementary Fig. 4). Overall, these results from primary mouse B cells and hybridomas showed that CSR can be achieved at high frequency by CRISPR-Cas9 system, and that hybridoma can be engineered to switch to the desired IgH subclass. Importantly, according to our strategy, CSR-edited hybridoma will retain the same V(D)J-coding sequence (Fig. 1a). Thus, this technology could be readily used to generate hybridoma with the same antigen specificity but different IgH subclass. This would represent an important application for antibody production because different IgH subclasses (for example, IgG_1_ versus IgG_4_) are known to have different affinity for the Fc receptor, potency in complement activation, biological properties in terms of half-life and tissue diffusion, as well different biochemical properties^16^.

### CRISPR-Cas9-mediated CSR in human B cells

**Figure 2:**
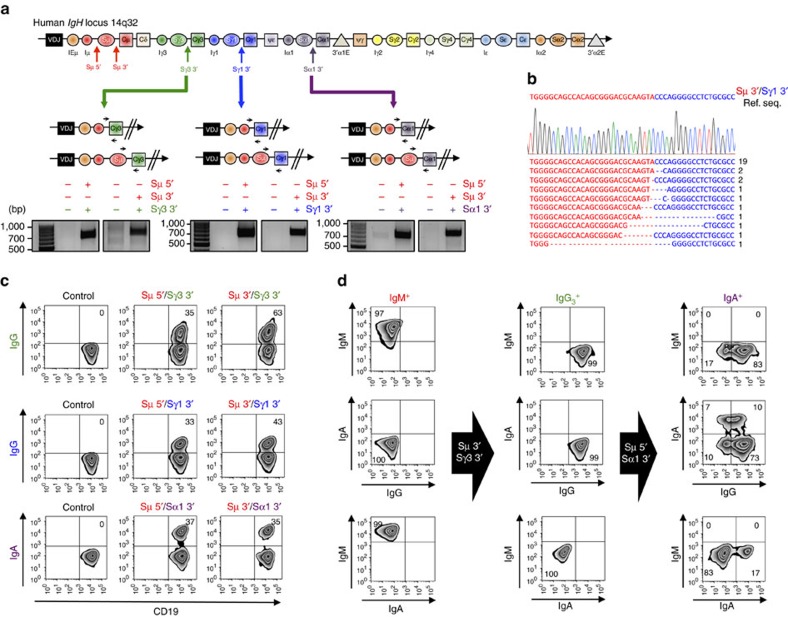
Induction of CSR by CRISPR-Cas9 system in human B cell lines. (**a**) Top: genomic organization of the human *IgH* constant region locus and position of the gRNAs. Bottom: schematic representation of six possible CSR products induced by deletions between Sμ and Sγ3, Sγ1 or Sα1 regions. Black arrows indicate the PCR primers designed to detect deletion. Gels show PCR amplicons obtained with the indicated primers. (**b**) Representative sequences of junctions identified from 30 clones for Sμ 3′ and Sγ1 3′ genomic region. Ref. Seq., sequence of the predicted genomic junction between Sμ 3′ and Sγ1 3′ region. Dashes: deleted bases. (**c**) IgM^+^ JEKO-1 cells were transduced with lentiviruses expressing Cas9 nuclease and gRNAs targeting Sμ and Sγ3, Sγ1 or Sα1 flanking regions. Cells were collected, co-stained with antibodies against CD19 and IgG or IgA, and analysed by flow cytometry. Representative zebra plots from three independent experiments are presented. Percentages of events are indicated in the corresponding quadrants. (**d**) To induce sequential CSR from IgM to IgG and then to IgA, IgM^+^ JEKO-1 cells were transduced with lentiviruses expressing Cas9 nuclease and gRNAs targeting Sμ 3′ and Sγ3 3′ flanking regions to generate IgG3^+^ JEKO-1 cells. IgG3^+^ cells were transduced again with lentiviruses expressing Cas9 nuclease and gRNAs targeting Sμ 5′ and Sα1 3′ flanking regions. Four days later, cells were collected, co-stained with IgM, IgG, or IgA antibodies and analysed by flow cytometry. Representative zebra plots from three independent experiments are presented. Percentages of events are indicated in the corresponding quadrants.

**Figure 3:**
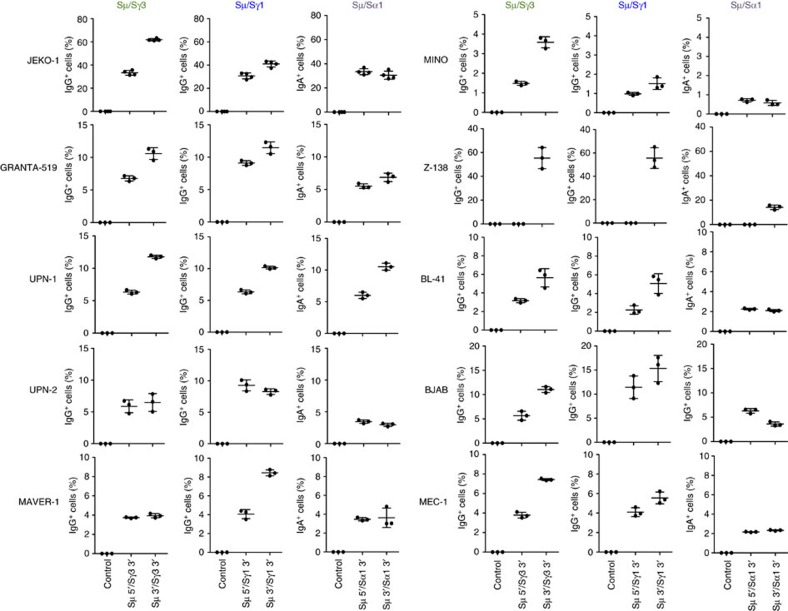
CSR in a panel of human B-cell lymphoma lines. Ten different human B-cell lymphoma lines (JEKO-1, GRANTA-519, UPN-1, UPN-2, MAVER-1, MINO, Z-138, BL-41, BJAB and MEC-1) were transduced with lentiviruses expressing Cas9 nuclease and gRNAs targeting Sμ and Sγ3, Sγ1, or Sα1 flanking regions. Two days later, cell lines were selected with puromycin (0.2 μg ml^−1^) for 3 days. Live cells were collected, co-stained with CD19 and IgG or IgA antibodies, and analysed by flow cytometry. As a control, non-transduced or single lentivirus-transduced cells were used. Data were analysed by FlowJo software. Mean±s.d.; *n*=3 biological replicates for each condition.

To investigate whether engineering of CSR could be efficiently achieved also in human B cells, we designed Cas9–gRNA lentiviral vectors to target the regions flanking the human Sμ (Sμ 5′ gRNA and Sμ 3′ gRNA), Sγ3 (Sγ3 3′ gRNA), Sγ1 (Sγ1 3′ gRNA) and Sα1 (Sα1 3′ gRNA) regions (Fig. 2a). We selected a panel of IgM^+^ human lymphoma cell lines that included mantle cell lymphoma (JEKO-1, GRANTA-519, UPN-1, UPN-2, MAVER-1, MINO and Z138), Burkitt lymphoma (BL-41 and BJAB) and chronic lymphocytic leukaemia (MEC-1). As shown in mouse B cells, deletion junctions were readily identified in all combinations tested and Sanger sequencing confirmed a prevalence of direct junctions over deletions and insertions (Fig. 2b; Supplementary Fig. 5). Excision circles and inversions were detected as well (Supplementary Figs 6 and 7). Remarkably, high levels of CSR were observed in each cell line tested ranging from 1% (MINO) to 60% (JEKO-1), with an average of 10% (Figs 2c and 3). The only lentivirus that did not induce CSR was Sμ 5′ gRNA in Z138 cell line. When we sequenced the DNA of Z138 corresponding to the gRNA target region, we found a small deletion encompassing the Sμ 5′ gRNA-targeted site, thus explaining why this gRNA combination failed to induce CSR. When we transduced B lymphoma cells with three gRNAs targeting the flanking regions of Sμ, Sγ1 and Sα, we simultaneously obtained IgG_1_^+^ and IgA^+^ cells from the original IgM^+^ cells at comparable frequency (Supplementary Fig. 8).

Next, we investigated whether human B cells could be engineered to undergo consecutive rounds of CSR, that is, whether B cells induced to switch first from IgM to IgG_3_ (or IgG_1_) were then editable to a sequential switch to IgA. Starting from IgM^+^ JEKO-1 cells, we first induced CSR to IgG_3_ or IgG_1_ by lentiviral transduction with Sμ 3′ gRNA and Sγ3 3′ gRNA or Sγ1 3′ gRNA and isolated pure IgG_3_^+^ or IgG_1_^+^ clones (Fig. 2d and Supplementary Fig. 9). Next, we transduced IgG_3_^+^ or IgG_1_^+^ clones with Sμ 5′ gRNA and Sα1 3′ gRNA to generate new DSBs in regions flanking Sμ and Sα. Remarkably, we obtained high levels of CSR to IgA, indicating that human B cells can be engineered to undergo multiple rounds of CSR at the same efficiency rate (Fig. 2d and Supplementary Fig. 9).

**Figure 4:**
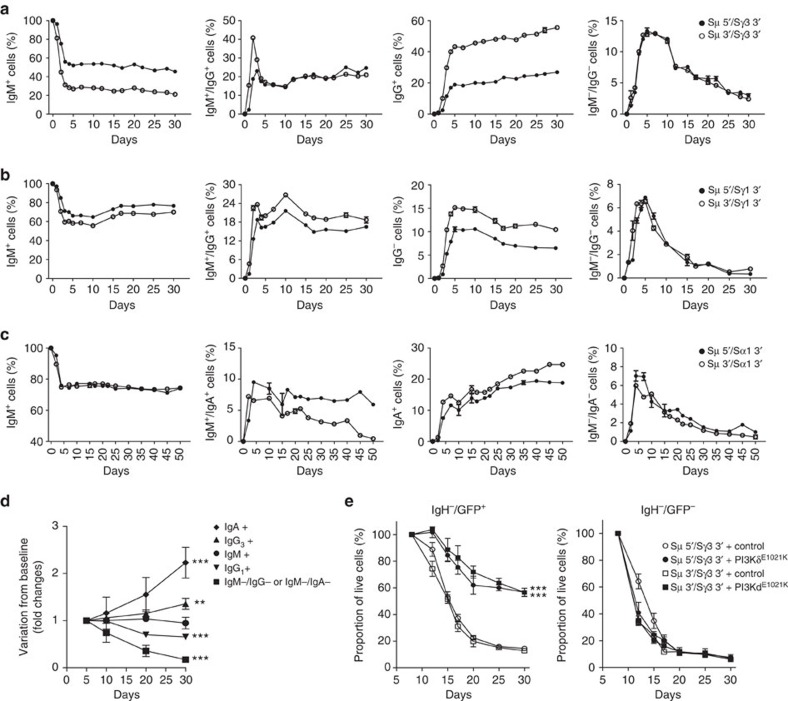
Biological effects of IgH subclass switch in human lymphoma cell growth. (**a**–**c**) To understand the growth of B cells after switch of the IgH subclass, JEKO-1 cells were co-transduced with two lentiviruses expressing Cas9 nuclease and gRNAs targeting Sμ, Sγ3 3′ (**a**), Sγ1 3′ (**b**) and Sα1 3′ (**c**) flanking regions. Cells were co-stained with antibodies against IgM and IgG or IgA and analysed by flow cytometry over time. (**d**) Statistical analysis of relative growth rates from lymphoma cells from Fig. 4a–c. Data are from at least four experiments in each condition. *P* values are calculated for each IgH subclass compared with native IgM^+^ cells. Data are expressed as mean ±s.d. for four independent experiments. Statistical analysis determined using two-way ANOVA (**P<0.01; ***P<0.001). (**e**) JEKO-1 cells were transduced with lentiviruses expressing Cas9 nuclease and gRNAs targeting Sμ and Sγ 3 regions. After 5 days, cells were transduced with retroviral vector expressing PI3Kδ^E1021K^ or control vector. Cells were co-stained with antibodies against IgM and IgG to allow the gating on the IgH-negative population (IgH^−^=IgM^−^/IgG^−^) and percentages of GFP^+^ cells were analysed over time by flow cytometry. As additional control IgH^-^ cells not transduced with the retrovirus (GFP^−^) were analysed. Data are from two independent experiments each repeated in duplicates and are expressed as mean±s.d. Statistical analysis determined using two-way ANOVA (***P<0.001).

The high efficiency of CSR obtained in human B cell allows for potential studies on the biological role of different IgH subclass in lymphoma biology. Signalling through the B cell receptor (BCR) is required to sustain survival and proliferation of normal B cells^17^,^18^, and drugs inhibiting the BCR signalling are effective in the treatment of B cell lymphoma^19^. However, it is not clear whether different IgH subclasses that recognize the same antigen can affect lymphoma growth, possibly through a different potency or quality of the BCR signalling. The efficiency and rapidity of our CRISPR-Cas9-based method for CSR opens the possibility to address these questions. As a proof of principle, we followed over time B lymphoma cells engineered to switch to different IgH subclasses. First, a small fraction of lymphoma cells transduced with Cas9–gRNA lost the expression of any IgH, likely due to large deletions around the Cas9 cleavage site or to non-coding inversions or translocations. These IgH-negative cells were selectively depleted over time, indicating a growth disadvantage of B lymphoma cells that lost the BCR signalling, as expected by previous studies^17^,^18^ (Fig. 4a-d). More surprisingly, different subclasses of IgH had contrasting biological effects on lymphoma growth, as IgG_1_^+^ cells had a significant growth disadvantage over IgM^+^ cells, whereas IgG_3_^+^ or IgA^+^ cells were positively selected (Fig. 4a-d), thus indicating that different IgH subclasses in B cell lymphoma have different biological properties.

Next, we further investigated whether loss of BCR signalling the lead to growth disadvantage in IgH-negative cells could be rescued by compensatory activation of key pathways downstream of the BCR signalling. This is an important biological concept because the BCR signalling is essential for the survival of malignant B cells through the activation of key downstream molecules such as the PI3Kδ pathway^18^. To this end, we took advantage of a newly discovered point mutation (PI3Kδ^E1021K^) that constitutively activates PI3Kδ independently of upstream BCR signalling and was recently described in patients with immunodeficiency and impaired CSR^20^,^21^. After induction of IgH loss in the JEKO-1 cell line by transduction with either Sμ 5′/Sγ3 3′ or Sμ 3′/Sγ3 3′ gRNAs, we subsequently transduced the lymphoma cells with lentivirus expressing GFP as reporter (control vector) or GFP and PI3Kδ^E1021K^. IgH-negative lymphoma expressing GFP were progressively depleted with similar kinetics to untransduced cells. In contrast, IgH-negative lymphoma cells expressing PI3Kδ^E1021K^ were significantly rescued as compared to GFP^-^ or GFP^+^ control cells (Fig. 4e).

### Generation of Fab′ fragment-secreting hybridomas

**Figure 5:**
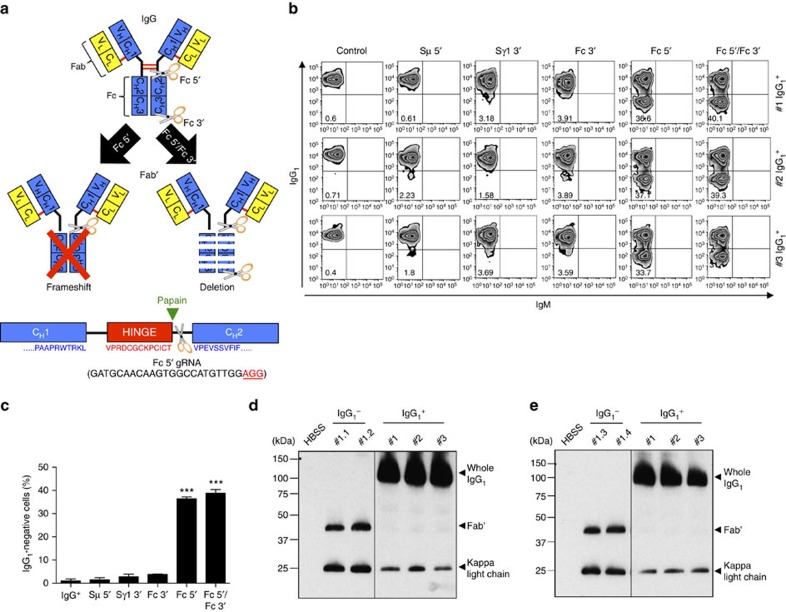
Generation of Fab′ fragments by CRISPR-Cas9 system in mouse hybridomas. (**a**) Top: schematic representation of mouse IgG antibody and target sites of two different gRNAs used for Fab′ fragment production. By using lentiviruses expressing Cas9 nuclease and Fc 5′ or Fc 5′/Fc 3′ guide RNAs, Fab′ fragments were produced by frameshift (left) or deletion (right) of the IgH Fc fragment, respectively. Bottom: schematic depiction of 5′ gRNA (scissors) in reference to the papain cleavage site. PAM sequence is underlined in red text. (**b**) IgG_1_^+^ hybridomas were transduced with lentivirus expressing Cas9 nuclease and gRNAs targeting Fc 5′ or Fc 5′ and Fc3′ regions. gRNAs targeting Sμ 5′, Sγ1 3′ or Fc 3′ were used as negative controls. Hybridomas were selected with puromycin, stained with IgM and IgG_1_ antibodies, and analysed by flow cytometry. Representative zebra plots from three different IgG_1_^+^ hybridomas are presented. Percentages of events are indicated in the corresponding quadrants. (**c**) Histograms representing the percentages of IgG-negative cells are shown with mean±s.d.; *n*=3 biological replicates for each condition. Statistical analysis determined using unpaired *t*-test (***P<0.001). (**d**,**e**) Western blot analyses of Fab′ fragments from hybridoma supernatants. IgG_1_^+^ hybridomas were transduced with lentiviruses expressing Cas9 nuclease and gRNAs targeting Fc 5′ or Fc 5′ and Fc3′ regions and IgG_1_-negative single clones were obtained by serial dilution. Examples of two clones from the frameshift approach (**d**; #1.1 and #1.2; purity >99%) and two clones from the deletion approach (**e**; #1.3 and #1.4; purity >99%) are shown. Supernatants were loaded on a SDS–PAGE in non-reducing condition, and developed with an anti-mouse kappa-light chain antibody. Three different IgG_1_^+^ hybridomas were used as controls.

Finally, the high efficiency of CRISPR-Cas9 system to edit the IgH locus prompted us to further investigate possible applications in antibody production. For several experimental or clinical applications, Fab′ fragments are preferable to whole antibodies. To obtain Fab′ fragments, purified antibodies are often processed by enzymatic digestion with proteases, such as papain or pepsin, followed by further purification to remove the Fc binding portion and to maintain the antigen-specific binding portion (Fab′ fragment)^22^. We reasoned that we could produce Fab′ fragments directly in hybridoma cells by deleting the Fc-coding sequence of the IgH chain. To obtain the Fc portion deletion, we designed gRNAs targeting the DNA proximal to the papain cleavage site of the IgG_1_ coding sequence. We tested either a frameshift approach where the deletion of the Fc portion is achieved by an out-of-frame NHEJ-mediated repair of the DSB introduced by Cas9 (Fc 5′) or a complete deletion approach where the DNA sequence for the Fc portion is deleted by two flanking DSBs (Fc 5′ and Fc 3′) (Fig. 5a). As a consequence of this deletion, the hybridoma should become IgH-negative because of the loss of the IgH membrane-binding domain^23^. Indeed, by either approach, we observed a high percentage of IgG_1_-negative cells (Fig. 5b,c), likely indicating an efficient deletion of the Fc fragment. To compare the relative efficiency of the two approaches we isolated single clones from a hybridoma co-transduced with Fc 5′ and Fc 3′. Out of the 64 isolated single-cell clones, only 4 (6.2%) deleted the Fc-coding sequence of the IgH chain, whereas in the majority of the clones the loss of the Fc coding was mediated by frameshift (Supplementary Figs 10 and 11). Next, we expanded IgG_1_-negative clones (Supplementary Fig. 12) and tested for production of Fab′ fragments. We collected hybridoma supernatants and separated the proteins on a SDS–PAGE gel in non-reducing conditions. By western blot assay with an anti-kappa-light chain antibody, IgG_1_-negative engineered hybridomas secreted Fab′ fragments together with the expected kappa-light chain^24^, whereas control IgG_1_^+^ hybridomas secreted the whole IgG_1_ as expected (Fig. 5d,e). As predicted, both the Fab′ fragments and the whole IgG_1_ completely disappeared when the SDS–PAGE gel was run in reducing conditions (Supplementary Fig. 13). Thus, hybridomas producing Fab′ fragments can be generated rapidly and effectively by CRISPR-Cas9 technology.

## Discussion

Altogether our data demonstrate the feasibility of a fast and efficient editing the Ig genes in human and mouse B cells by CRISPR-Cas9 technology. CSR was induced with equal efficiency whether the gRNAs were directed to target sequences upstream or downstream the switch regions, as predicted by the current models of CSR^1^,^25^. Consistent with the notion that CSR is a peculiar form of gene rearrangement in the *IgH* locus, when we introduced two gRNAs in the cells we found additional genomic rearrangements, such as inversions and interchromosomal translocations as we and others previously described with CRISPR-Cas9 technology^11^,^12^,^13^,^14^. The predominance of precise junctions between the two DSBs generated by Cas9 reflects the described property of Cas9 to generate blunt ends 3 bp upstream of the PAM sequence^26^. Blunt ends are then joined by the c-NHEJ pathway, which could also be responsible for small deletions or insertions^4^.

Efficiency of CSR was remarkably high in hybridoma and human B lymphoma cells. This was quite surprising but consistent with the observations made by our group and others that rearrangements occur with high frequency between two DSBs that occur at a low genomic distance on the same chromosome^5^,^15^,^27^,^28^. The lower CSR achieved in primary mouse B cells was likely due to a lower efficiency of transduction than hybridomas and human B cells. Currently used lentiviral or retroviral CRISPR-Cas9 vectors produce relatively low viral titres due to the large size of the constructs^29^. The recent demonstration that smaller Cas9 molecules obtained by *Staphylococcus aureus* have similar efficiency to *Streptococcus pyogenes* (SpCas9) allows the design of smaller retroviral constructs that should generate more efficient viruses^30^. In addition, the cloning of two gRNAs in the same vector instead of two independent vectors should further increase the efficiency of CSR by increasing the number of cells co-expressing the two gRNAs.

CRISPR-Cas9-mediated CSR in hybridoma cells was efficient, fast and easy allowing for CSR to any desired IgH subclass. Implication for the technology of antibody production could be profound. For example, IgG antibodies are preferred for applications such as western blot analysis, immunohistochemistry and ELISA, whereas IgM clones are typically discarded because IgM are pentameric, more difficult to purify and less stable than IgG. In addition, different IgG subclasses have different stability, as well as biological and biochemical properties. For examples, effector functions in terms of triggering FcγR-expressing cells, activating complement, phagocytosis or antibody-dependent cell-mediated cytotoxicity are different within IgG_1_, IgG_2_, IgG_3_ and IgG_4_ subclasses^30^. By applying the approach we propose, it would be possible to easily switch hybridomas to the desired IgH subclass for any laboratory or therapeutic use. In addition, we have also shown that hybridoma can be engineered to produce Fab′ fragments instead of the corresponding whole IgH molecule. Fab′ fragments were directly produced and secreted by hybridoma cells in culture at comparable levels to the unedited whole IgH molecules. This approach would largely simplify the method for the production of Fab′ fragments that is currently based on several steps of protease cleavage followed by purification of the resulting fragments. We propose that engineering hybridomas to directly produce Fab′ fragments will represent an important advancement given that Fab′ fragments are preferred for several experimental approaches as well as for therapeutic deliver of more effective antibody–drug conjugates^31^.

Finally, CSR was achieved in human B cells at high efficiency, a feat never accomplished so far. In contrast to primary mouse B cells, induction of CSR in human B cells is largely inefficient with current methods^32^. In contrast, by CRISPR-Cas9 technology we were able to rapidly and efficiently induce CSR in all human B-cell lines tested to any desired IgH subclass. We propose that Cas9-mediated editing provides a highly useful approach for the study of the functional activity of different IgH subclass in the physiology and pathology of human B cells as the contribution of the different IgH subclasses is largely unknown. Furthermore, unveiling the network of signalling pathways initiated by the BCR has become increasingly important to understand the physiology of normal B cells^17^ as well as the pathological survival and expansion of neoplastic B cells^19^. Indeed, inhibition of BCR activity has recently changed the treatment landscape for B-cell malignancies^33^,^34^,^35^,^36^,^37^,^38^. Inhibitors of key molecules in BCR signalling such as PI3Kδ inhibitors (idelalisib) or Bruton tyrosine kinase-BTK inhibitors (ibrutinib) have been recently approved by the FDA for the treatment of CLL or MCL^39^ and others are under investigation^40^. As we showed in reconstitution experiments with activated PI3Kδ constructs, modelling CSR or eliminating BCR signalling by CRISPR-Cas9 technology will provide opportunities to precisely reconstruct the contribution of single pathways or molecules to the biology of normal and neoplastic B cells.

## Methods

### Animals

Female 129/SvJ, 129S2 and AID-deficient mice at 8-12 weeks of age were used in the experiments. A minimum of three mice for each experiment was used. No exclusion criteria were used. 129S2 mice (Charles River) and AID-deficient mice, which were kindly provided by Dr Frederick W. Alt (Boston Children's Hospital), were housed and maintained in the specific pathogen free (SPF) facility at Boston Children's Hospital. Animal experiments were performed under protocol approved by the Institutional Animal Care and Use Committee (IACUC) of Boston Children's Hospital (Protocol #13-01-2295).

### Plasmid DNA constructions

For SpCas9 expression and generation of guide-RNA (gRNA), the 20-nucleotide target sequences were selected to precede a 5′-NGG protospacer-adjacent motif (PAM) sequence. To minimize off-target effects, the CRISPR design tool from Dr Feng Zhang laboratory was used (http://crispr.mit.edu/). All gRNA and PAM sequences used in this study are listed in Supplementary Table 1. Oligonucleotides were purchased from Integrated DNA technology (IDT), annealed and cloned into the *BsmBI*–*BsmBI* sites downstream from the human U6 promoter in LentiCRISPR v2 plasmid, which was a gift from Dr Feng Zhang (Addgene plasmid #52961). Oligonucleotides used in this study for cloning are listed in Supplementary Table 2.

To generate RetroCRISPR v1 plasmid, all gRNAs were first cloned in LentiCRISPR v2 vector. To obtain retroviral backbone, pMSCVgfp::AID plasmid, a gift from Nina Papavasiliou (Addgene plasmid # 15925), was digested with XhoI and EcoRI restriction enzymes to remove AID gene, and repaired by Klenow fragment. The constructs harbouring the U6 promoter–gRNA–gRNA scaffold-EF1α promoter-SpCas9-NLS-flag-P2A-Puro were obtained from LentiCRISPR v2 vector, and repaired by Klenow fragment. By ligating those two constructs, we generated RetroCRISPR v1 plasmid (Supplementary Fig. 3a). Since smaller size of plasmid is more efficient to produce retroviral particles, we decided to make a RetroCRISPR v2 plasmid. The construct harbouring BamHI-P2A-GFP-ClaI was PCR amplified using pMSCVgfp:AID as a template with forward primer: 5′-TAAGGGATCCGGCGCAACAAACTTCTCTCTGCTGAAACAAGCCGGAGATGTCGAAGAGAATCCTGGA CCGGTGAGCAAGGGCGAGGAGCTGTTC-3′ and reverse primer: 5′-TAAGATCGATGGCCGCTTTACTTGTACAGCTCGTCCATGC-3′ (BamHI and ClaI sites are underlined). To generate RetroCRISPR v2 plasmid, PCR products were cloned into RetroCRISPR v1 plasmid digested with BamHI and ClaI restriction enzymes (Supplementary Fig. 3b).

PI3Kδ^E1021K^ construct cloned in GFP-reporter MIGR1 retroviral vector was a kind gift from Dr Klaus Okkenhaug (The Babraham Institute, Cambridge, UK). As a control, we used GFP-reporter MIGR1 plasmid, which was a gift from Warren Pear (Addgene plasmid # 27490).

### Lenti- and Retro-viral particle productions

HEK293FT cells (Invitrogen), Phoenix-ECO cells (ATCC), and GP2-293 packaging cells (Clontech) were maintained in DMEM supplemented with 10% fetal bovine serum (FBS; ATLANTA), 100 units per ml penicillin–streptomycin (P/S; Corning), and 2 mM L-Glutamine (L-Glu; Corning). Cells were cultured at 37 °C in 5% CO_2_ atmosphere. The cell lines were tested and resulted negative for *Mycoplasma* contamination.

To generate lentiviral particle, 5.5 × 10^6^ HEK293FT cells were plated per 10-cm dish. The following day, cells were transfected by calcium phosphate transfection method (CalPhos Mammalian Transfection Kit; Clontech) with 7.2 μg of lentiCRISPR plasmid, 3.6 μg of pMD2.G (Addgene plasmid #12259), 3.6 μg of pRSV-Rev (Addgene plasmid #12253) and 3.6 μg of pMDLg/pRRE (Addgene plasmid #12251). The media was changed 8 h post-transfection. The viral supernatant was collected 48 h post-transfection, passed through a 0.45-μm filter, pooled and used either fresh or snap frozen.

To generate retroviral particle for mouse B cells, 3.5 × 10^6^ Phoenix-ECO cells were plated per 10-cm dish. The following day, cells were transfected by calcium phosphate transfection method (CalPhos Mammalian Transfection Kit) with 10 μg of retroviral plasmid and 5 μg of pCL-Eco retrovirus packaging plasmid. The media was changed 8 h post-transfection. The viral supernatant was collected 48 h post-transfection, passed through a 0.45-μm filter, pooled and used either fresh or snap frozen.

To generate retroviral particle for human cells, 3.5 × 10^6^ GP2-293 packaging cells were plated per 10-cm dish. The following day, cells were transfected by Xfect transfection reagent (Clontech) with 10 μg of retroviral plasmid and 5 μg of pCMV-VSV-G (Addgene plasmid #8454) retrovirus envelop plasmid. The media was changed 4 h post-transfection. The viral supernatant was collected 48 h post-transfection, passed through a 0.45-μm filter, pooled and used either fresh or snap frozen.

### Cell cultures, transduction and puromycin selection

Mouse fibroblast cells immortalized with SV-40 LT (pBABE-puro SV40 LT; Addgene plasmid # 13970) were maintained in DMEM supplemented with 10% FBS, 100 units per ml P/S, and 2 mM L-Glu. Cells were cultured at 37 °C in 5% CO_2_ atmosphere. For transduction, 2 × 10^4^ cells were plated into six-well plates. The following day, cells were transduced with viral supernatant supplemented with 6 μg ml^−1^ polybrene. The viral supernatant was exchanged for fresh medium 6 h later. After 2 days, cells were treated with 6 μg ml^−1^ of puromycin to select resistant cells until non-infected cells were completely dead.

Naive B cells were separated from total spleen cell suspensions using anti-CD43 magnetic microbeads (Miltenyi). The CD43-negative fraction was cultured with anti-CD40 antibody (1 μg ml^−1^; eBioscience) and IL-4 (20 ng ml^−1^; PeproTech) for 4 days. Retrovirus infection was performed 24 h post-activation. Activated-B cells were transduced with viral supernatant supplemented with 6 μg ml^−1^ polybrene. The viral supernatant was exchanged for fresh medium containing anti-CD40 antibody and IL-4 6 h later.

Hybridomas were generated by fusion between anti-CD40 and IL-4-stimulated B cells from 129/SvJ mice and NS-1 fusion partner myeloma cells on day 4 and were recovered after 7 days selection with 1 × Hypoxanthine Aminopterin Thymidine (HAT) medium. Single clones from each well were picked and screened by ELISA on their supernatants with IgM and IgG_1_ capturing and revealing antibodies (Southern Biotech). Only clones that were single positive for one of the two antibody classes were used in this study. Hybridomas were cultured in RPMI 1640 medium GlutaMax (Invitrogen) supplemented with 15% FBS and 100 units per ml P/S. Hybridomas were maintained at 37 °C in 5% CO_2_ atmosphere. For transduction, 2 × 10^5^ cells were plated into six-well plates and transduced with viral supernatant supplemented with 6 μg ml^−1^ polybrene. The viral supernatant was exchanged for fresh medium 6 h later. After 2 days, cells were treated with 3 μg ml^−1^ of puromycin to select resistant cells until non-infected cells were completely dead.

Mantle cell lymphoma (JEKO-1, GRANTA-519, MAVER-1 and MINO from DSMZ; Z-138 from ATCC; UPN-1 and UPN-2 from Dr Alberto Zamo' (University of Verona, Italy), Burkitt lymphoma (BL-41 and BJAB from DSMZ), and chronic lymphocytic leukaemia (MEC-1 from DSMZ) cell lines were used in this study. All human lymphomas were cultured and maintained in RPMI 1640 medium supplemented with 10% FBS, 100 units per ml^−1^ P/S, and 2 mM L-Glu. All cell lines were cultured at 37 °C in 5% CO_2_ atmosphere. The cell lines all tested negative for *Mycoplasma* contamination. For transduction, 2 × 10^5^ cells were plated into six-well plates and transduced with viral supernatant supplemented with 6 μg ml^−1^ polybrene. The viral supernatant was exchanged for fresh medium 6 h later. After 2 days, cell lines were treated with 0.2 μg ml^−1^ of puromycin to select resistant cells until non-infected cells were completely dead. For PI3Kδ^E1021K^ rescue experiments, JEKO-1 cells were first transduced with lentiviruses to induce deletion between Sμ and Sγ3, cultivated for 5 days and then transduced with GFP-reporter PI3Kδ^E1021K^ or control MIGR1 retrovirus.

### Genomic DNA isolation, PCR and sequencing analysis

Mouse fibroblast, hybridomas and JEKO-1 cells were transduced with lentiviruses, selected with puromycin and collected after 5 days of transduction. Genomic DNA was extracted using Rapid lysis buffer (100 mM Tris-HCl pH8.0, 200 mM NaCl, 5 mM EDTA, 0.2% SDS) with 100 μg ml^−1^ Proteinase K by incubating at 56 °C overnight. Genomic DNA was precipitated in one volume isopropanol, and the DNA pellet was resuspended in 10 mM Tris-HCl (pH 8.0). Primers used for PCR amplifications to detect deletions, inversions, and excision circles from mouse fibroblasts, hybrodomas or JEKO-1 cells are listed in Supplementary Table 3. PCR products were gel purified and cloned using pGEM-T easy vector system (Promega). Mutations were identified by Sanger sequencing. Uncropped gel images are shown in Supplementary Figs 14 and 15.

### Surveyor assay

The genomic region flanking the CRISPR target sites was PCR amplified (Surveyor primers are listed in Supplementary Table 3), and products were purified using PCR purification kit (QIAGEN) following the manufacturer's protocol. A total of 400 ng of the purified PCR products were mixed with 2 μl 10 × Taq DNA Polymerase PCR buffer (Life Technologies) and ultrapure water to a final volume of 20 μl, and subjected to a reannealing process to enable heteroduplex formation: 95 °C for 10 min, 95 °C to 85 °C ramping at −2 °C per second, 85 °C to 25 °C at −0.25 °C per second, and 25 °C hold for 1 min. After reannealing, products were treated with SURVEYOR nuclease and SURVEYOR enhancer S (Transgenomics) for 1 h at 42 °C, and analysed on 2% high-resolution agarose gel (A4718, Sigma Aldrich). Gels were stained with ethidium bromide (Sigma Aldrich) and imaged with a Gel Doc gel imaging system (Bio-rad). Quantification was based on relative band intensities. Indel percentage was determined by the formula, 100 × (1−(1−(*b*+*c*)/(*a*+*b*+*c*))1/2), where a is the integrated intensity of the undigested PCR product, and *b* and *c* are the integrated intensities of each cleavage products.

### Flow cytometry

Mouse B cells and hybridomas were stained with PE-conjugated anti-IgG_1_ antibody (Clone A85-1, 550083, BD Pharmingen, 1:100 dilution) or APC-conjugated anti-IgM antibody (17-5790-82, eBioscience, 1:100 dilution) for 30 min on ice, and analysed using a FACSVerse flow cytometer (BD Biosciences). IgG_1_^+^ cells were gated on GFP. Data were analysed by FlowJo software.

Human lymphoma cells were co-stained with APC-conjugated anti-CD19 (Clone 4G7-2E3, FAB4867A, R&D System, 1:100 dilution) and either FITC-conjugated anti-IgM (AHI1608, MyBioSource, 1:100 dilution) or FITC-conjugated anti-IgG (AHI1308, MyBioSource, 1:100 dilution) or FITC-conjugated anti-IgA antibodies (AHI1108, MyBioSource, 1:100 dilution) for 30 min on ice to detect IgG or IgA switching, respectively. For simultaneous switching, JEKO-1 cells were transduced with three different lentiviruses indicated in Supplementary Fig. 9. Cells were co-stained with PE-conjugated anti-IgG (AHI1307, MyBioSource) and FITC-conjugated anti-IgA antibodies (MyBioSource) for 30 min on ice. To detect sequential switching, cells were stained with the three following combinations: FITC-conjugated anti-IgM and PE-conjugated anti-IgG, FITC-conjugated anti-IgA and PE-conjugated anti-IgG, or PE-conjugated anti-IgM (AHI1607, MyBioScience, 1:100 dilution) and FITC-conjugated anti-IgA antibodies for 30 min on ice. Cells were analysed using a FACSVerse flow cytometer (BD biosciences). Data were analysed by FlowJo software (FlowJo).

### Western blot analysis

IgG_1_- or Fab′ fragment-producing hybridomas were cultured in six-well plates (3 × 10^6^ cells in 2 ml of HBSS). After 24 h, supernatants were collected and centrifuged to remove dead cells. For non-reducing condition, supernatants were mixed with loading buffer without reducing reagents. For reducing condition, supernatants were mixed with loading buffer containing β–mercaptoethanol and boiled at 95 °C for 5 min. In both conditions, samples were loaded on 4-15% Mini-PROTEIN TGX gels (BIO-RAD), transferred on nitrocellulose membrane (GE Healthcare), blocked with 5% Skim milk (BIO-RAD), incubated with Rat monoclonal anti-mouse kappa-light chain (HRP) (clone H139-52.1, ab99632, Abcam, 1:5,000 dilution) or Goat polyclonal anti-mouse IgG-H&L chain (HRP) (NA931V, GE Healthcare, 1:5,000 dilution), and developed with ECL solution (GE Healthcare). Uncropped western blot images are shown in Supplementary Figs 16 and 17.

### Statistical analysis

The statistical analysis represented mean±s.d. from three or more independent experiments. Data were analysed by unpaired *t*-test for group differences and by two-way analysis of variance analysis of variance for condition and group differences together using GraphPad Prism 6 software; **P<0.01, ***P<0.001.

## Additional information

**How to cite this article:** Cheong, T.-C. *et al.* Editing of mouse and human immunoglobulin genes by CRISPR-Cas9 system. *Nat. Commun.* 7:10934 doi: 10.1038/ncomms10934 (2016).

## Supplementary Material

Supplementary Information Supplementary Figures 1-17 and Supplementary Tables 1-3
