# Supplementary material for: Editing of mouse and human immunoglobulin genes by CRISPR-Cas9 system
Source: Nat Commun. 2016 Mar 9;7:10934. doi: 10.1038/ncomms10934 (PMC4786874; doi:10.1038/ncomms10934)
Supplement: Supplementary Information — Supplementary Figures 1-17 and Supplementary Tables 1-3 [file ncomms10934-s1.pdf]

Supplementary Figure 1

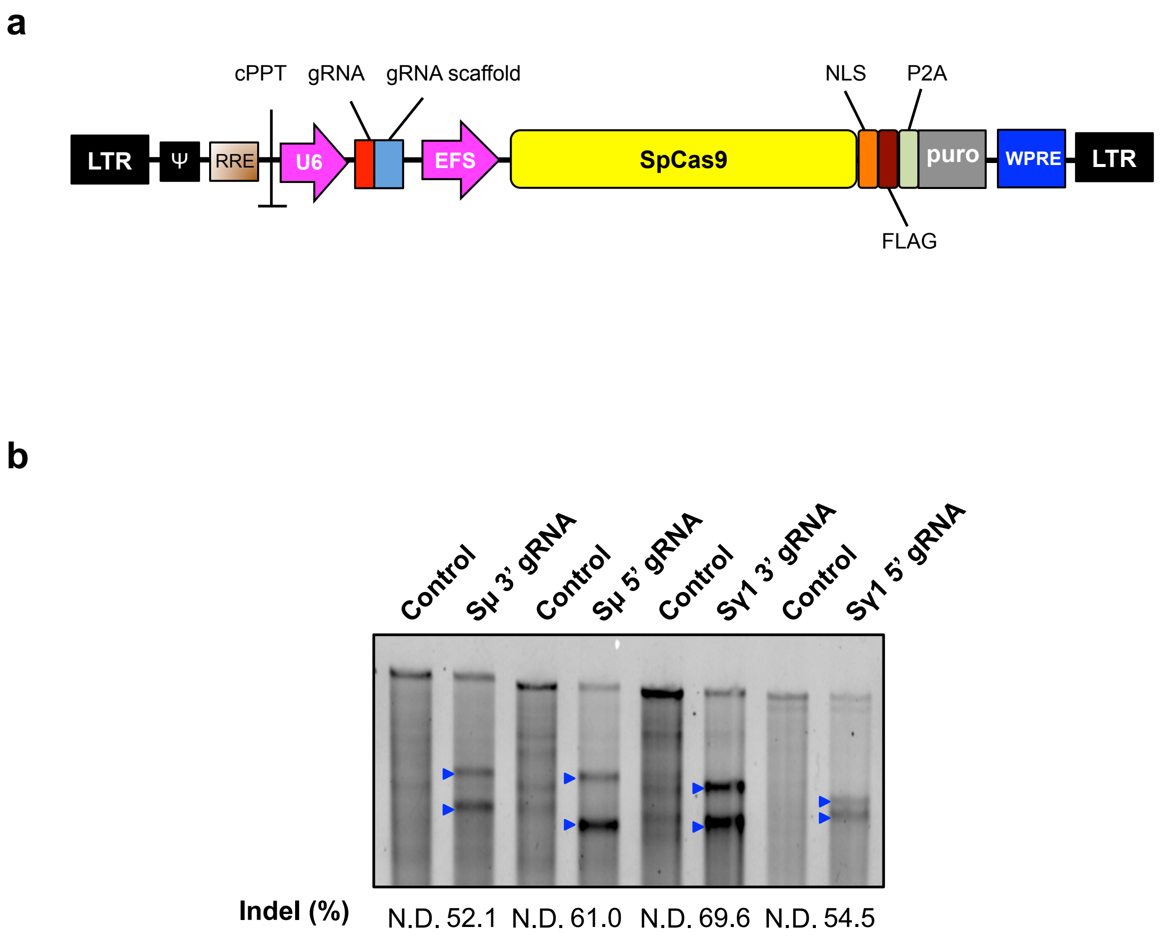

Supplementary Figure 1. LentiCRISPR v2 vector and surveyor assay. (**a**) Schematic overview of lentiCRISPR v2 vector (addgene #52961). (**b**) Evaluation of the efficiency of gRNAs targeting in mouse fibroblast cells. Locus modification efficiencies were analyzed 5 days after transduction using Surveyor nuclease assay. Estimated indel formation is indicated below each target. Blue arrowheads indicate expected bands. N.D.: not detected.

Supplementary Figure 2

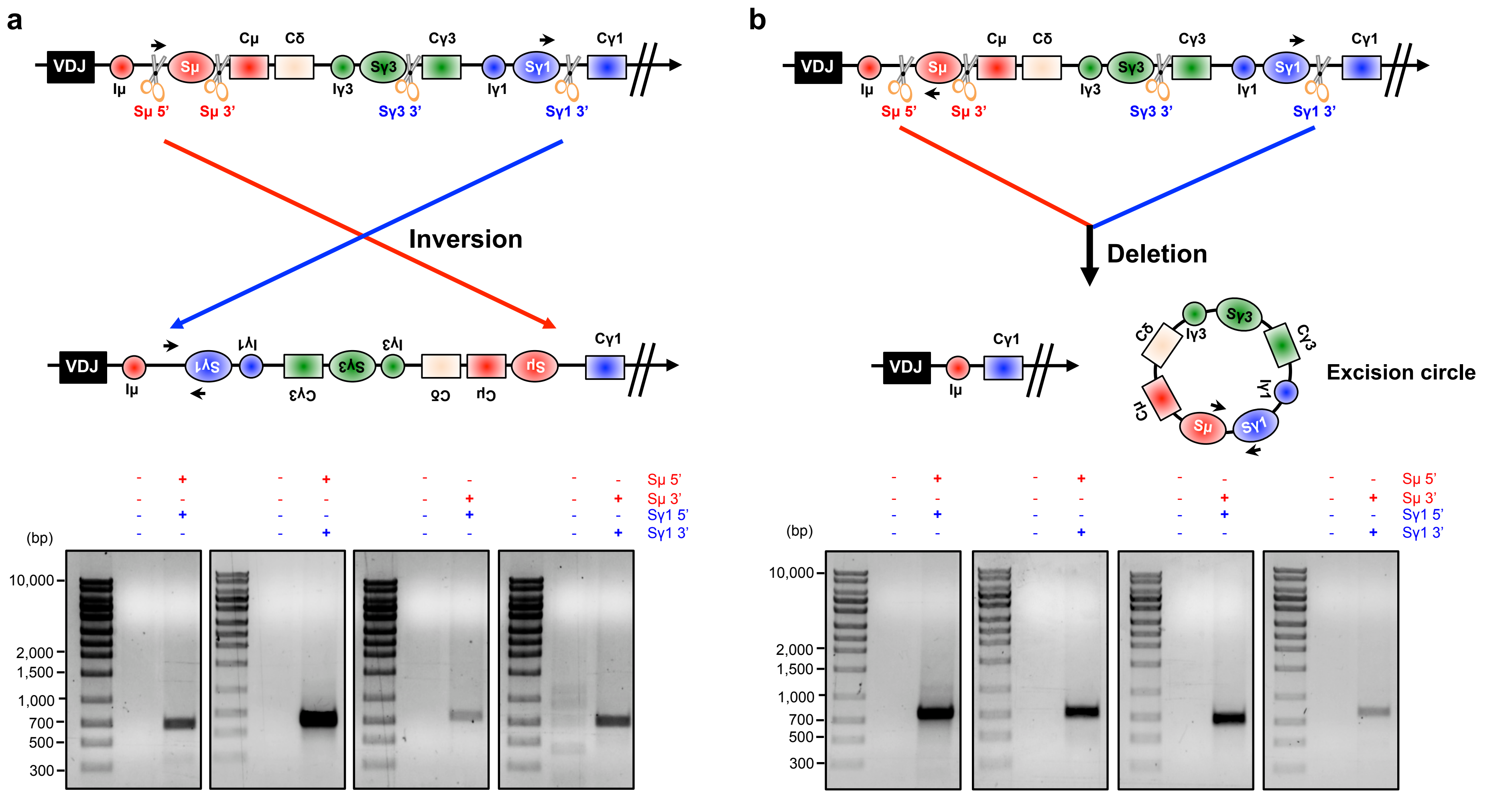

Supplementary Figure 2. Detection of inversions and excision circles. (**a**) Top: Schematic overview of an example of inversion between Sμ 5' and Sγ1 3'. Black arrows indicate primers for PCR. Cutting sites are indicated by scissors. Bottom: Detection of deletions by PCR on genomic DNA extracted from mouse fibroblast transduced with two lentiviruses expressing Cas9 nuclease and gRNAs targeting Sμ and Sγ1 flanking regions. Mouse fibroblast cells were transduced with lentiviruses, selected with puromycin (6 μg ml^{-1}), and collected at day 7 to isolate genomic DNA. (**b**) Top: Schematic overview of an example of excision circle between Sμ 5' and Sγ1 3'. Black arrows indicate primers for PCR. Cutting sites are indicated by scissors. Bottom: Detection of excision circles by PCR on genomic DNA extracted from mouse fibroblast transduced with two lentiviruses expressing Cas9 nuclease and gRNAs targeting Sμ and Sγ1 flanking regions. Mouse fibroblast cells were transduced with lentiviruses, selected with puromycin (6 μg ml^{-1}), and collected at day 7 to isolate genomic DNA.

Supplementary Figure 3

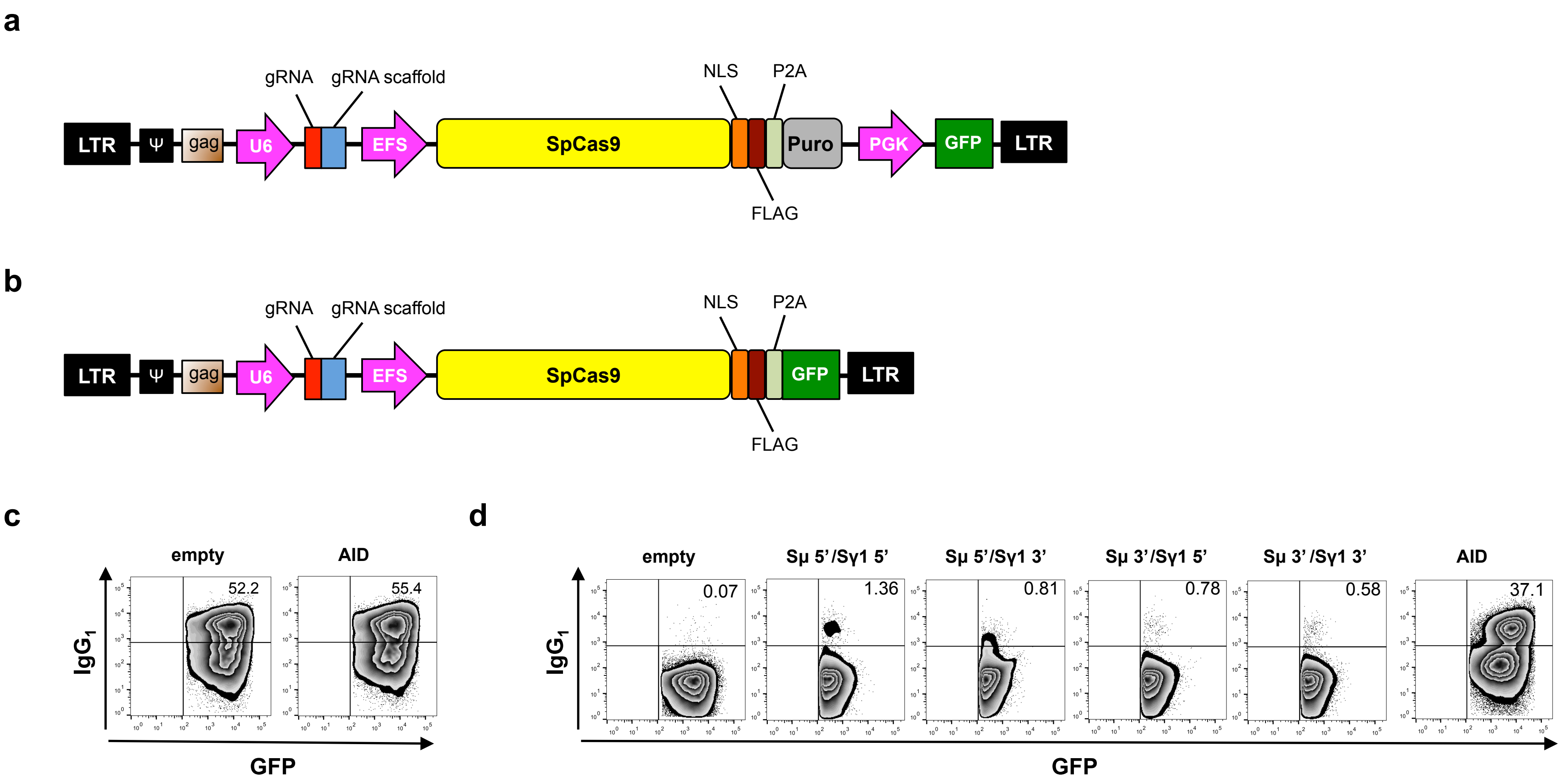

Supplementary Figure 3. RetroCRISPR vectors and detection of CSR in mouse splenic B cells. (**a**) Schematic overview of RetroCRISPR v2 vector. (**b**) Schematic overview of RetroCRISPR v2 vector. pMSCVgfp::AID vector (addgene #15925) was cut to obtain retroviral backbone and ligated with the segment from U6 promoter to puromycin isolated from LentiCRISPR v2 (**Supplementary Figure 1**). GFP was amplified by PCR with primers, and replaced with puromycin in the vector. (**c-d**) Untouched mouse B cells isolated from the spleen of 129S2 WT (**c**) and AID-deficient (**d**) mice were activated by anti-CD40 antibody and IL-4 for 1 day and then transduced with retrovirus expressing Cas9 nuclease and gRNAs used in (**b**). GFP expressing-retrovirus or AID expressing-retrovirus was used as negative or positive control, respectively. Three days later, cells were harvested, stained with IgG_1 antibody, and then analyzed by flow cytometry. IgG_1^+ cells were gated on the GFP positive population. Representative zebra plots are presented and percentages of events are indicated in the corresponding quadrant.

Supplementary Figure 4

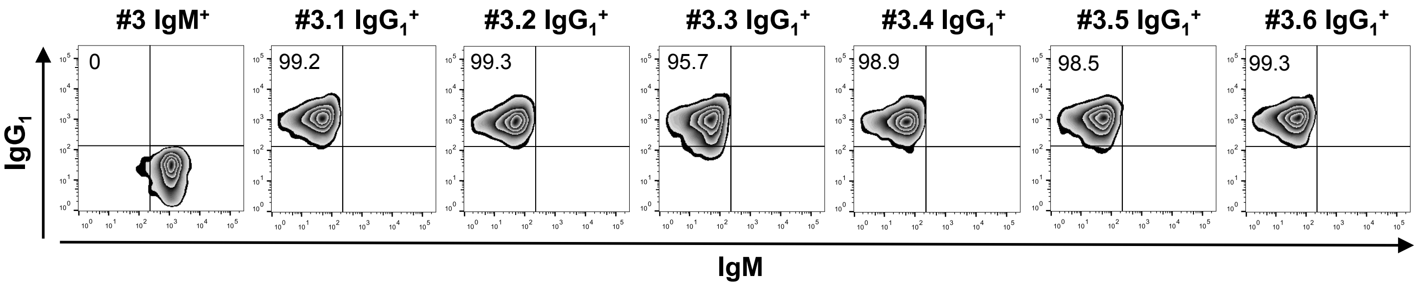

Supplementary Figure 4. Examples of CSR in hybridomas. Hybridoma #3 IgM$^+$ from **Fig. 1d** was transduced with four different combinations of lentiviruses expressing Cas9 nuclease and gRNAs used in **Fig. 1a** and selected with puromycin (3 μg ml^{-1}) for 3 days. Live cells were seeded in 96-well plates to isolate single cell clones, stained with IgG_1 and IgM antibodies, and analyzed by flow cytometry. Data were analyzed by FlowJo software. Out of 189 hybridomas, 12 were IgG_1-positive pure clones. Representative zebra plots from six different IgG_1-positive pure clones are presented. Percentages of events are indicated in the corresponding quadrant.

Supplementary Figure 5

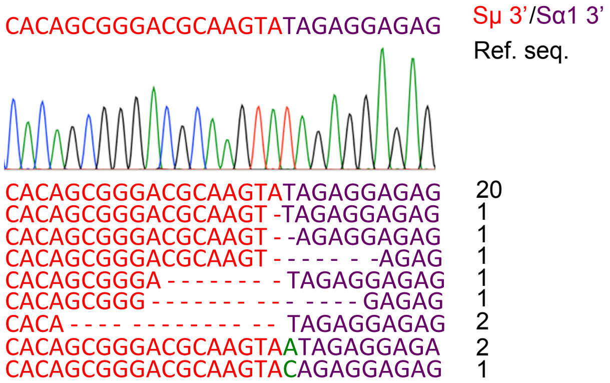

Supplementary Figure 5. Examples of Sanger sequencing of junction between Sm and Sa flanking regions. Representative sequences of junctions identified from 30 clones for Sµ 3' and Sa1 3' genomic region. PCR products were purified and cloned into pGEM-T vector. Ref. Seq., sequence of the predicted genomic junction between Sµ 3' and Sa1 3' region. Green: insertions; Dashes: deleted bases.

Supplementary Figure 6

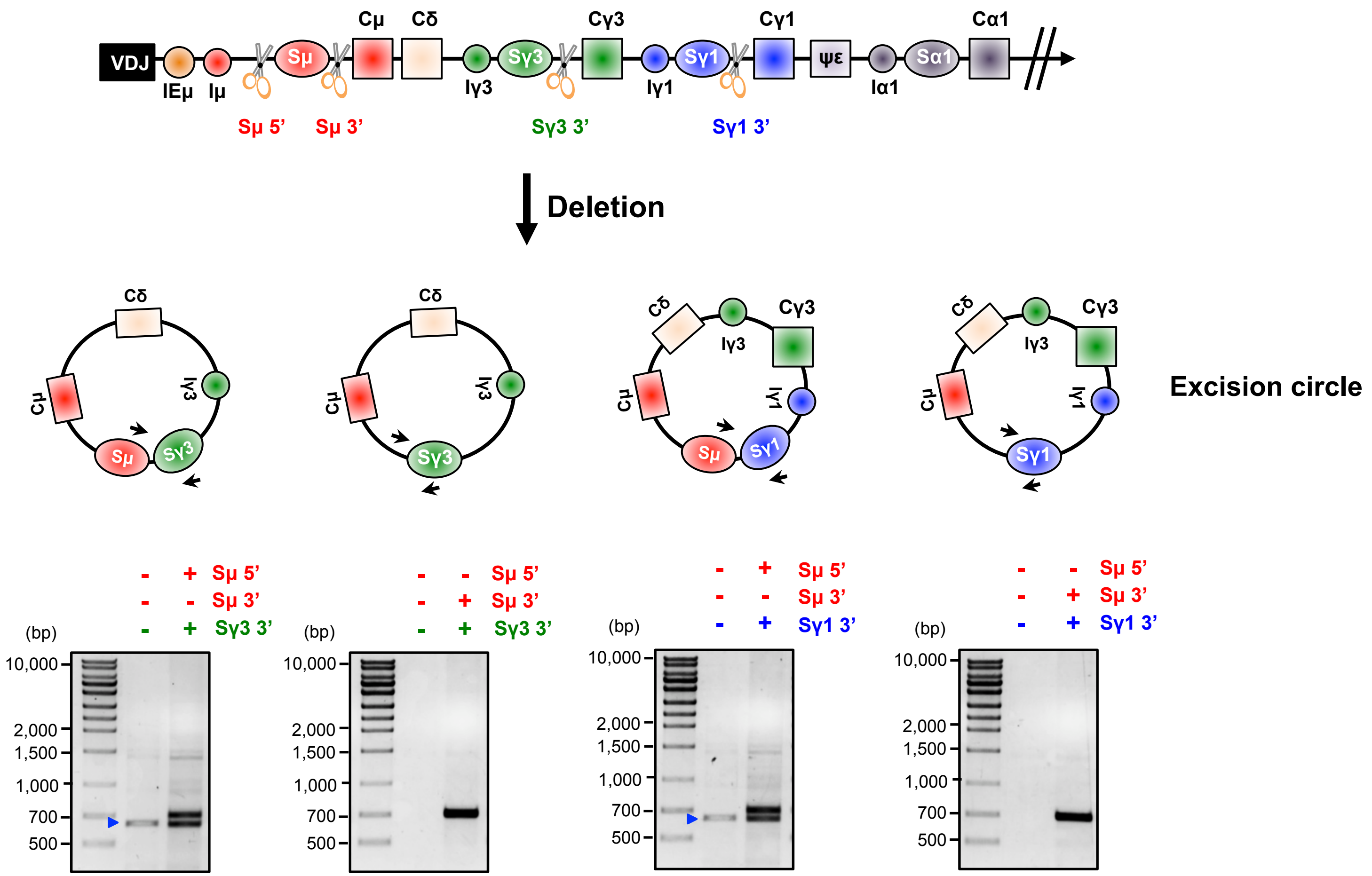

Supplementary Figure 6. Detection of excision circles in JEKO-1 cells. Schematic overview of excision circles generated with four different combinations of gRNA targeting Sμ and Sγ3 or Sγ1 flanking regions. Black arrows indicate primers used for PCR. Cutting sites are indicated with scissors. Blue arrowheads indicate non-specific band.

Supplementary Figure 7

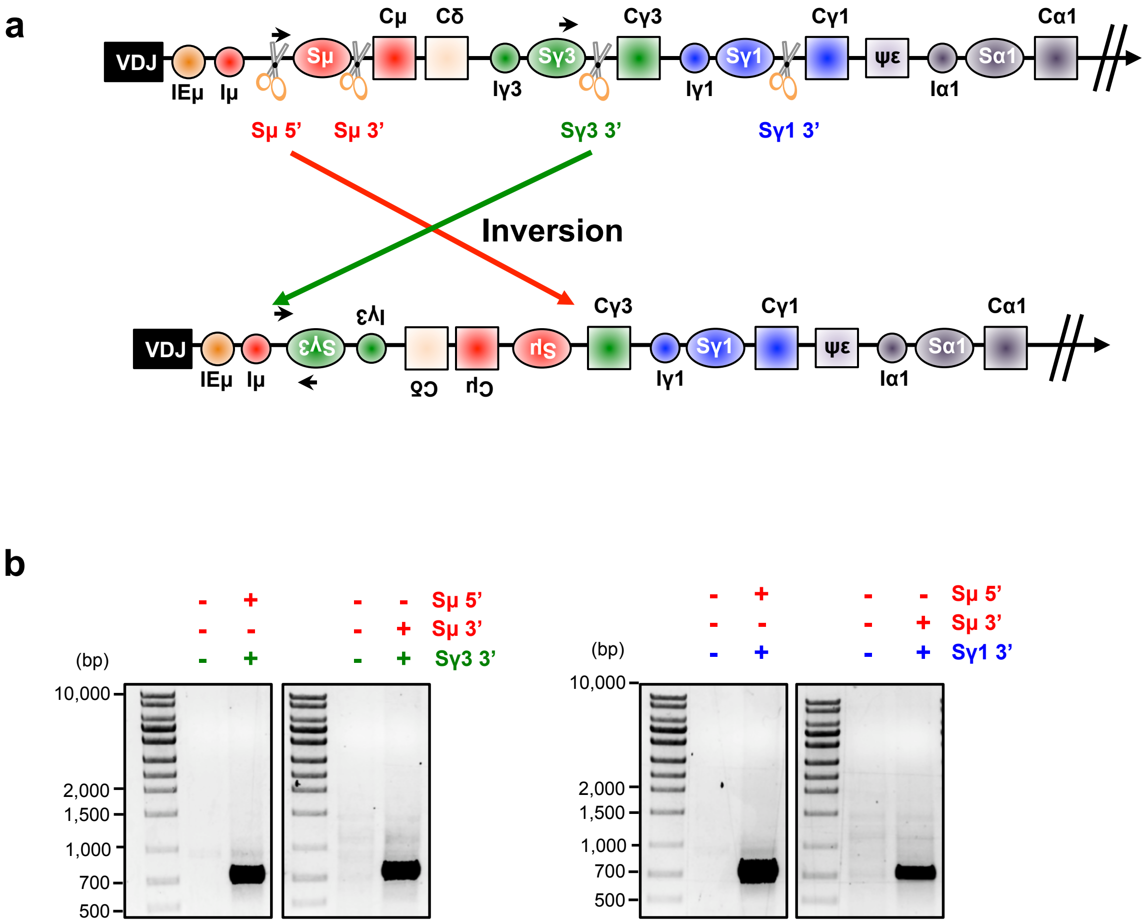

Supplementary Figure 7. Detection of inversions in JEKO-1 cells. (**a**) Schematic overview of an example of an inversion resulting from gRNA targeting Sμ 5' and Sγ3 3' flanking regions. Black arrows indicate primers for PCR. Cutting sites are indicated with scissors. (**b**) Detection of excised circle by PCR on genomic DNA extracted from JEKO-1 cells transduced with two lentiviruses expressing Cas9 nuclease and gRNAs targeting Sμ and Sγ3 or Sγ1 flanking regions.

Supplementary Figure 8

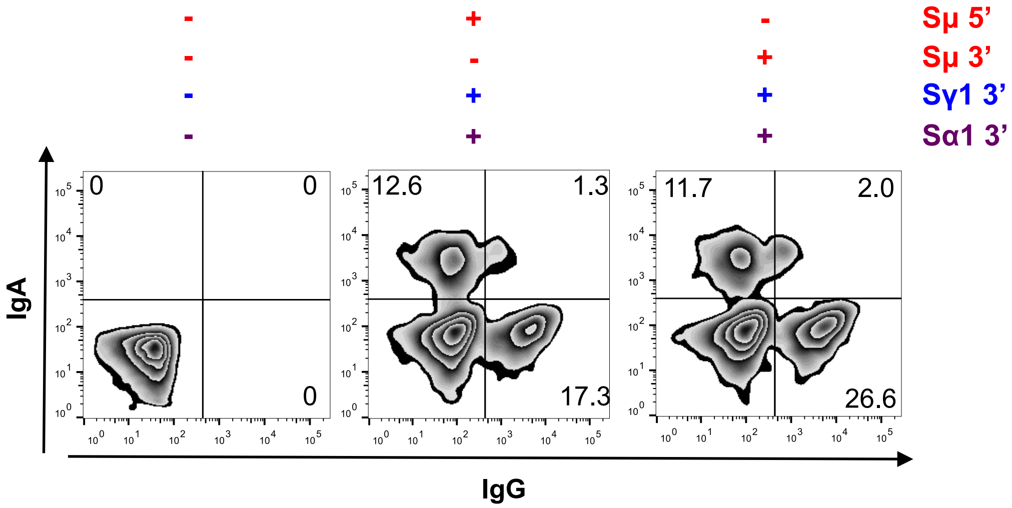

Supplementary Figure 8. Simultaneous CSR switching in JEKO-1 cells. To induce simultaneous class switching from IgM to IgG or IgA, IgM-expressing JEKO-1 cells were co-transduced with three lentiviruses expressing Cas9 nuclease and gRNAs targeting Sμ, Sγ1 3', and Sα1 3' flanking regions. Two days later, cells were selected with puromycin (0.2 μg ml^{-1}). At day 7, cells were collected, co-stained with antibodies IgM, IgG, or IgA, and analyzed by flow cytometry. Data were analyzed by FlowJo software. Representative zebra plots from one of three independent experiments are presented. Percentages of events are indicated in the corresponding quadrants.

Supplementary Figure 9

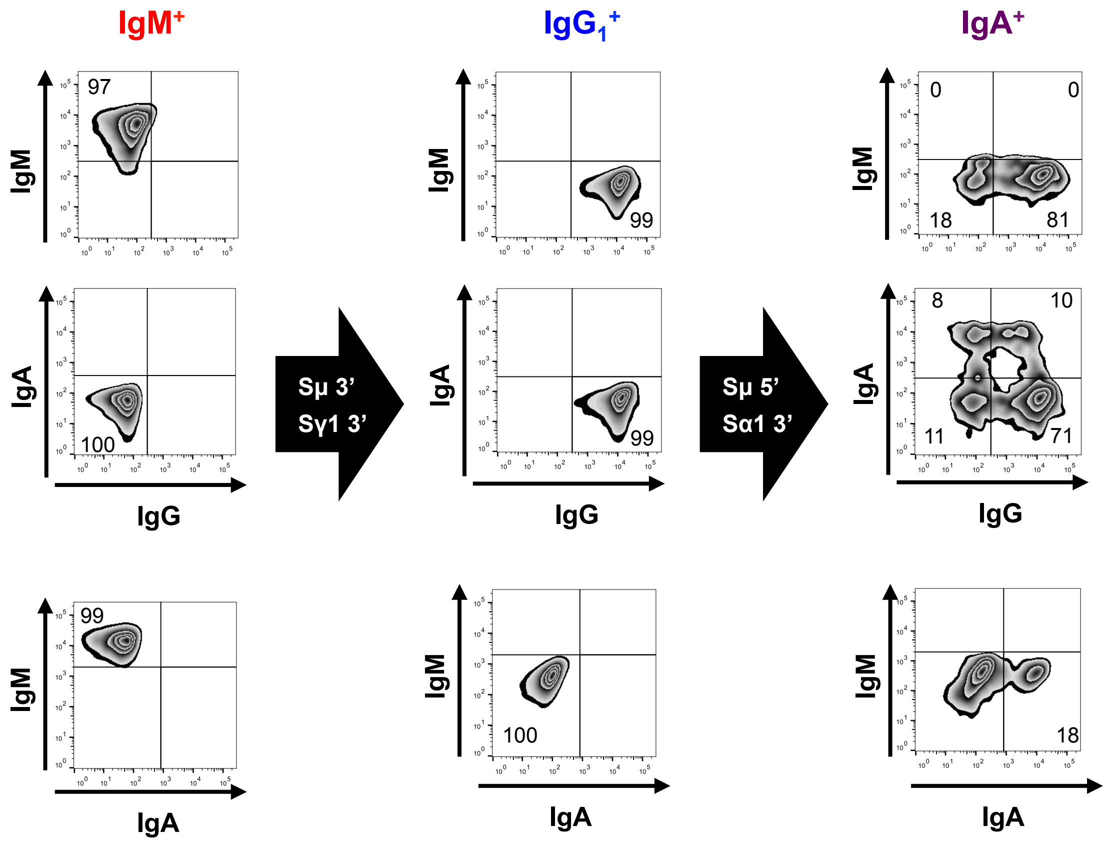

Supplementary Figure 9. Sequential CSR in JEKO-1 cells. To induce sequential class switching from IgM to IgG and then to IgA, IgM-expressing JEKO-1 cells were transduced with lentiviruses expressing Cas9 nuclease and gRNAs targeting Sμ 3’ and Sγ1 3’ flanking regions to generate IgG_1-expressing JEKO-1 cells. IgG_1-expressing cells were then transduced with lentiviruses expressing Cas9 nuclease and gRNAs targeting Sμ 5’ and Sα1 3’ flanking regions. Four days later, cells were collected, co-stained with antibodies IgM, IgG, or IgA, and analyzed by flow cytometry. Data were analyzed by FlowJo software. Representative zebra plots from one of three independent experiments are presented. Percentages of events are indicated in the corresponding quadrants.

Supplementary Figure 10

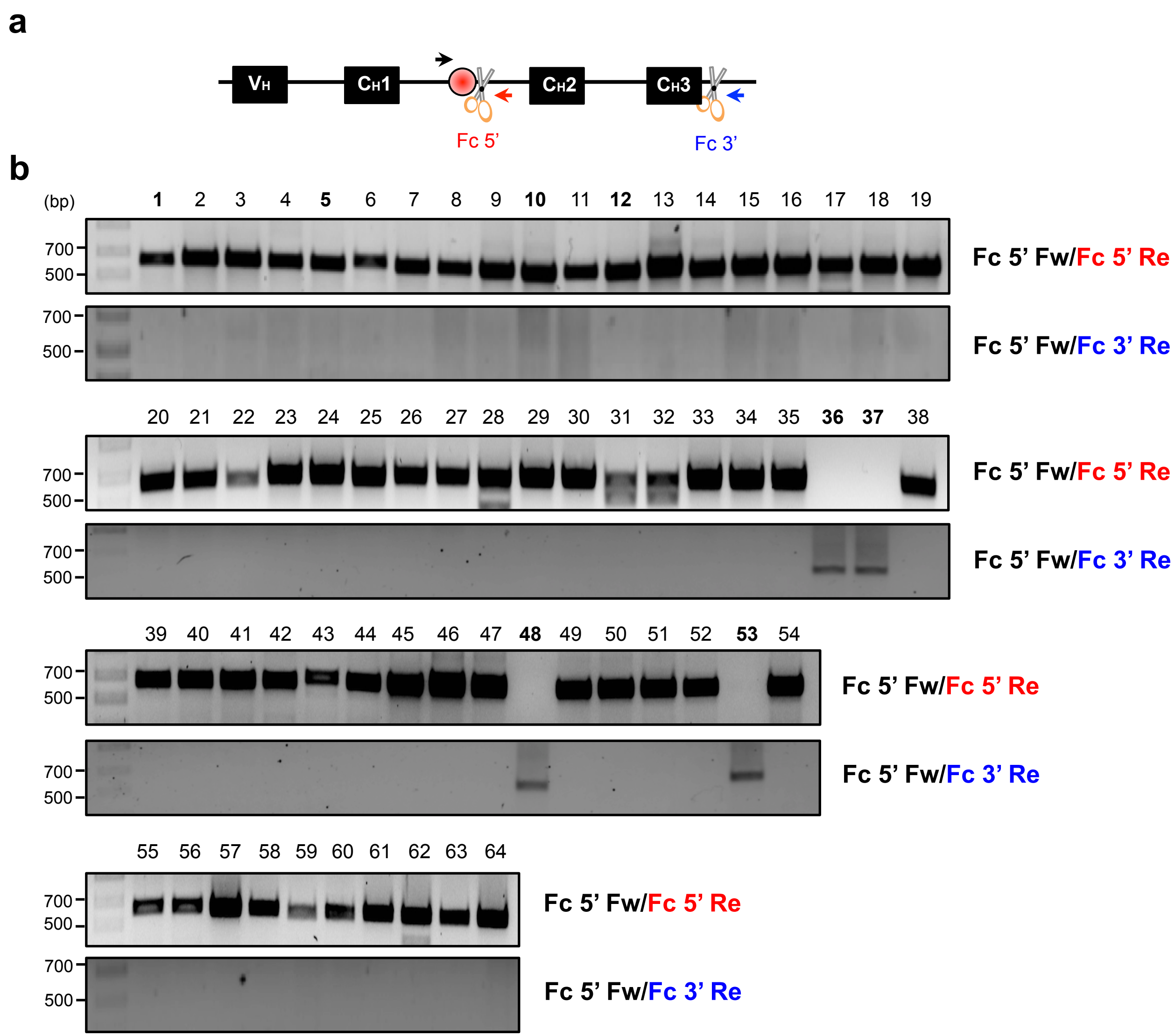

Supplementary Figure 10. Detection of frameshift vs deletion frequency in Fab' producing hybridomas. (a) Schematic overview of the PCR approach to distinguish between frameshift or deletion mediated removal of IgH Fc portion in single cell clones obtained from IgH negative hybridomas. Black arrow indicates primer for Fc 5' forward primer. Red and blue arrows indicate primers for Fc 5' reverse and Fc 3' reverse, respectively. Red circle represents IgH hinge region. (**b**) PCR reactions were performed with the indicated primer sets (colour coded). Four (#36, #37, #48 and #53) out of 64 clones showed deletion of the IgH Fc portion.

Supplementary Figure 11

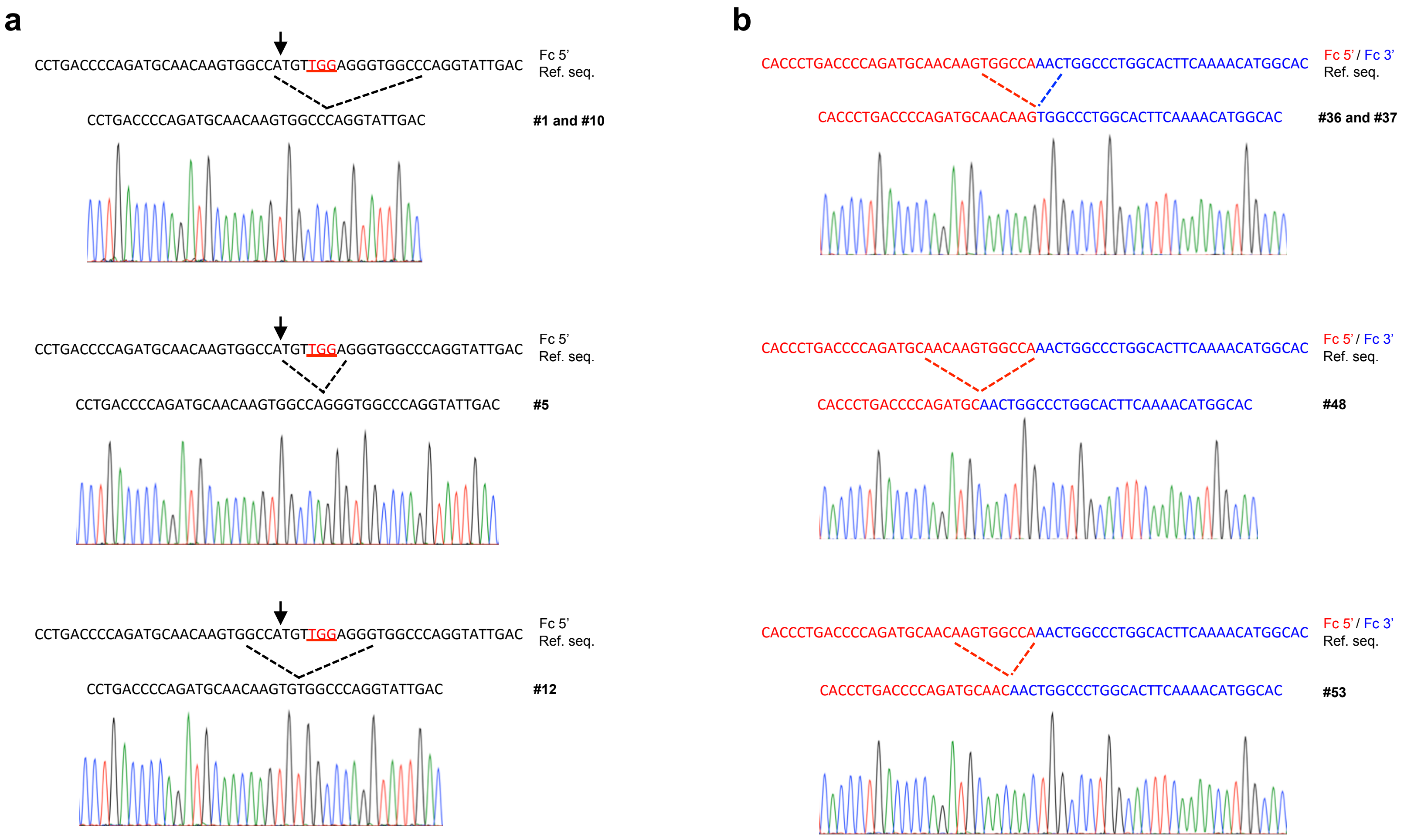

Supplementary Figure 11. Examples Sanger sequencing of junctions in Fab' producing hybridomas with frameshift or deletion of the IgH Fc portion. (**a**) Examples Sanger sequencing of junctions of Fc 5' region in Fab' hybridomas with frameshift mediated removal of the IgH Fc portion. Ref. Seq., sequence of the predicted genomic junction of Fc 5' region. Black arrow indicates Cas9 cutting site. PAM sequence is underlined in red. (**b**) Examples Sanger sequencing of junctions in Fab' hybridomas with deletion mediated removal of the IgH Fc portion. Ref. Seq., sequence of the predicted genomic junction between Fc 5' and Fc3' regions.

Supplementary Figure 12

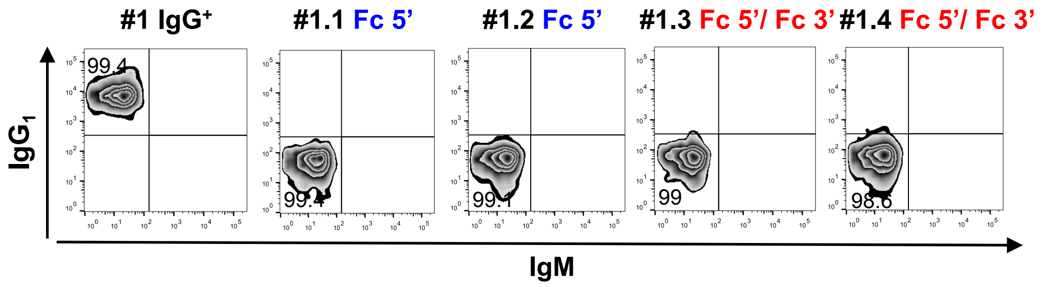

Supplementary Figure 12. Generation of hybridomas producing Fab’ fragments. IgG_1-expressing hybridoma (#1 from **Fig. 5b**) was transduced with lentivirus expressing Cas9 nuclease and gRNAs targeting Fc 5’ (frameshift) or Fc 5’ and Fc3’ together (deletion). Cells were selected with puromycin (3 μg ml^{-1}) for 3 days, and cultured in 96-well plates to isolate IgG_1-negative single cell clones expressing only Fab’ fragments. Hybridomas were stained with IgM and IgG_1 antibodies, and analyzed by flow cytometry. Two clones from the frameshift approach (#1.1 and 1.2) and 2 clones from deletion approach (#1.3 and #1.4) were generated with purity >99%. Data were analyzed by FlowJo software. Representative zebra plots are presented. Percentages of events are indicated in the corresponding quadrant.

Supplementary Figure 13

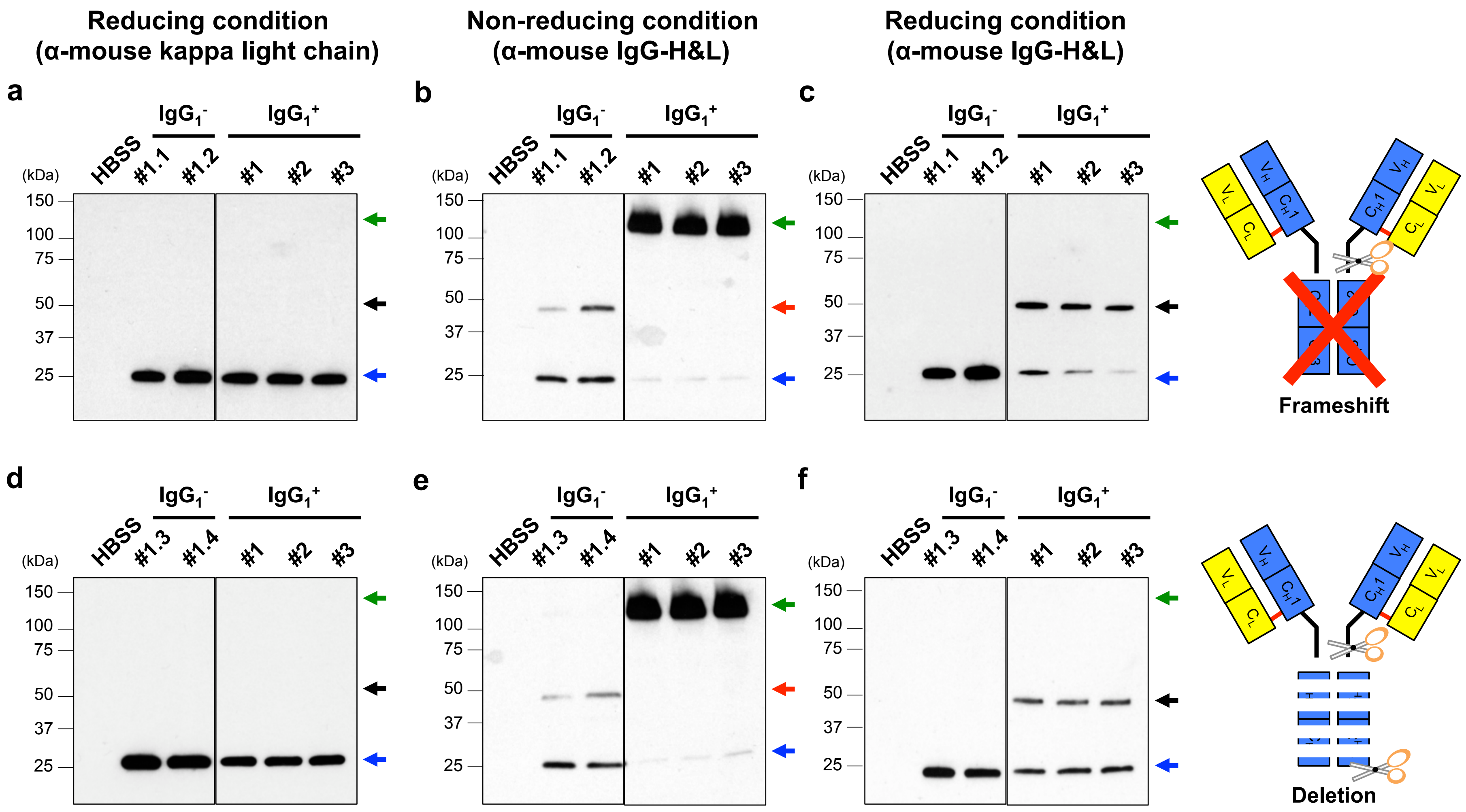

Supplementary Figure 13. Detection of Fab' fragments in the supernatants of hybridomas. Western blot analyses of supernatants collected from hybridomas. IgG_1-expressing hybridomas were transduced with lentiviruses expressing Cas9 nuclease and gRNAs targeting Fc 5' (frameshift) or Fc 5' and Fc 3' together (deletion). Two days later, cells were treated with puromycin (3 μg ml^{-1}) to select IgG_1-negative hybridomas. Two clones from the frameshift approach (#1.1 and 1.2) and 2 clones from deletion approach (#1.3 and #1.4) as in **Supplementary Fig. 12** were isolated and cultured in HBSS for 1 day. Supernatants were collected, loaded on the 4-15% gradient SDS-PAGE with reducing agent (β-mercaptoethanol; **a, c, d,** and **f**) or in non-reducing conditions (**b** and **e**) and developed with an anti-mouse kappa light chain antibody (**a** and **d**) or an anti-mouse IgG H&L antibody (**b, c, e,** and **f**). Blue arrows: kappa light chain; Red arrows: Fab' fragments; Green arrows: whole IgG_1; Black arrow: IgG_1 heavy chain.

Supplementary Figure 14

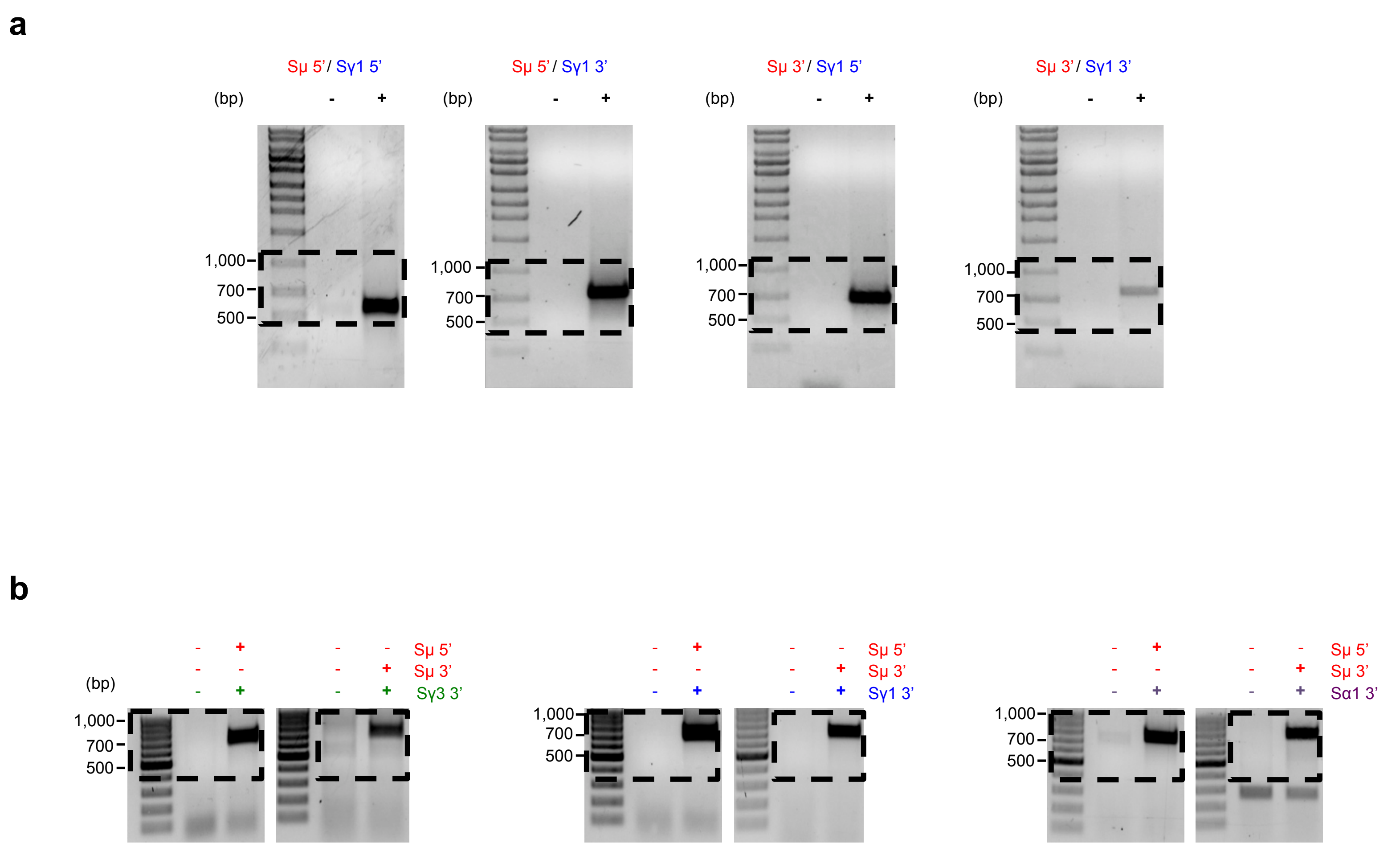

Supplementary Figure 14. Uncropped scans. (a) Corresponds to Figure 1a. (b) Corresponds to Figure 2a.

Supplementary Figure 15

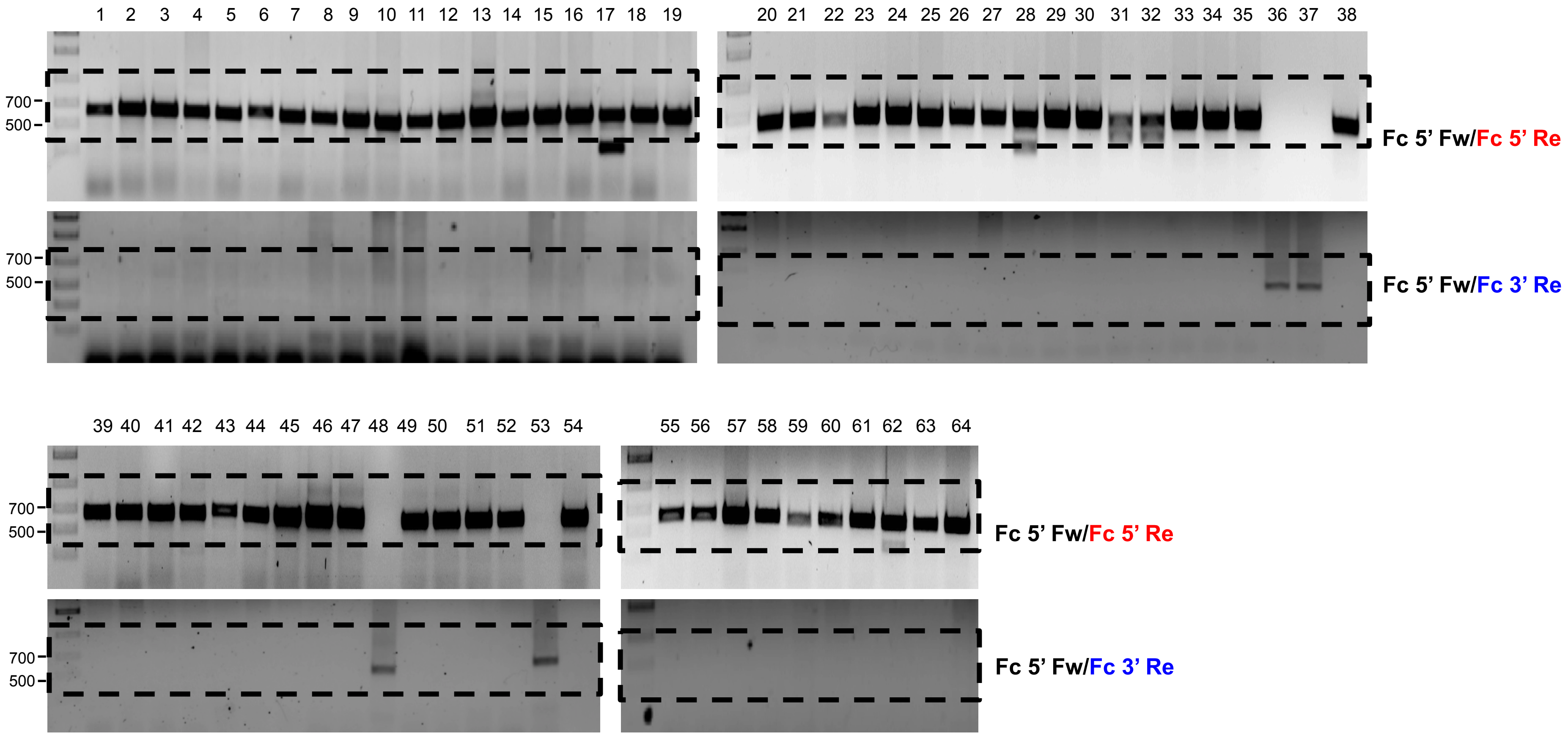

Supplementary Figure 15. Uncropped scans. (a-f) Corresponds to Supplementary Fig. 10b.

Supplementary Figure 16

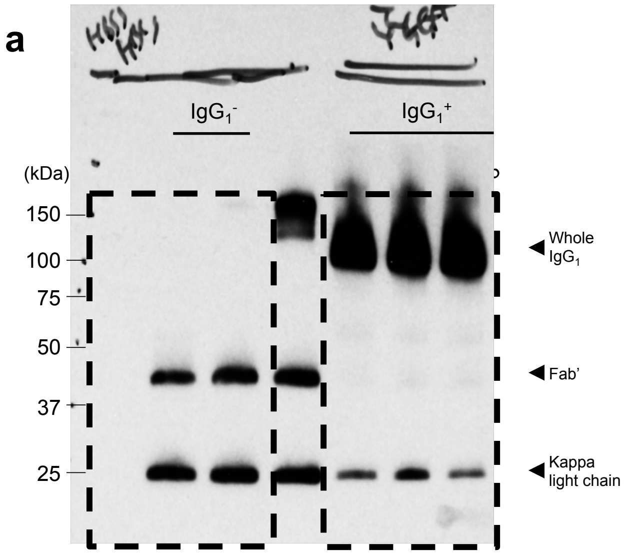

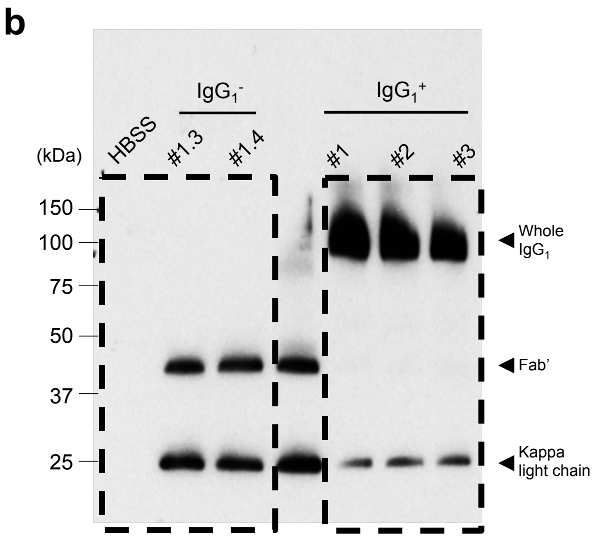

Supplementary Figure 16. Uncropped scans. (a) Corresponds to Figure 5d. (b) Corresponds to Figure 5e.

Supplementary Figure 17

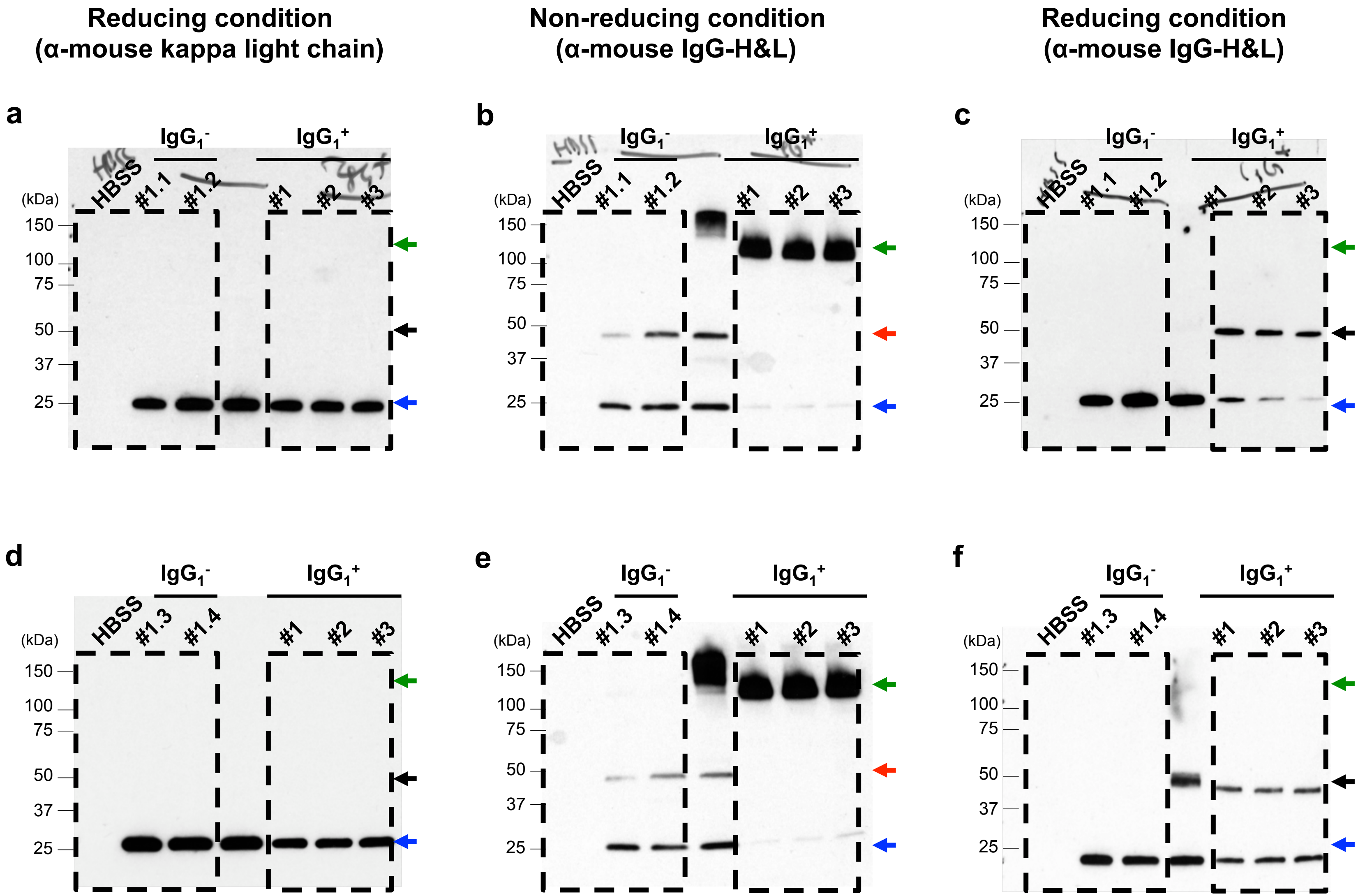

Supplementary Figure 17. Uncropped scans. (a-f) Corresponds to Supplementary Fig. 13.

Supplementary Table 1. gRNAs and PAM sequences used in this study.

Target	20nt-guide	PAM
**Mouse IgH**		
Sμ 5’	GCCAGAGGCAGCCACAGCTG	TGG
Sμ 3’	GTCACGTTCCTGTGGCTAGA	AGG
Sγ1 5’	GGAAAGTGCAAGCTGCTCTG	AGG
Sγ1 3’	GAGAGTCGGGGACATGGGAA	GGG
**Hybridoma**		
Fc 5’	GATGCAACAAGTGGCCATGT	TGG
Fc 3’	TGTGCTCTTCCTATGCAAAC	TGG
**Human IgH**		
Sμ 5’	ATATTCCACCCAGGTAGTGG	AGG
Sμ 3’	ACAGCGGGACGCAAGTAGTG	AGG
Sγ3 3’	TCGTGGATAGACAAGAACCG	AGG
Sγ1 3’	TCGCGGACAGTTAAGAACCC	AGG
Sα1 3’	GCTGGGTTCCTCCAGTATAG	AGG

Supplementary Table 2. Oligonucleotides cloned in CRISPR-Cas9 lenti- or retro-viral vectors.

Primer	Sequence (5'- 3')
**Mouse IgH**	
Sμ 5' Fw	CACCGCCAGAGGCAGCCACAGCTG
Sμ 5' Re	AAACCAGCTGTGGCTGCCTCTGGC
Sμ 3' Fw	CACCGTCACGTTCCTGTGGCTAGA
Sμ 3' Re	AAACTCTAGCCACAGGAACGTGAC
Sγ1 5' Fw	CACCGGAAAGTGCAAGCTGCTCTG
Sγ1 5' Re	AAACCAGAGCAGCTTGCACTTTCC
Sγ1 3' Fw	CACCGAGAGTCGGGGACATGGGAA
Sγ1 3' Re	AAACTTCCCATGTCCCCGACTCTC
**Hybridoma**	
Fc 5' Fw	CACCGATGCAACAAGTGGCCATGT
Fc 5' Re	AAACACATGGCCACTTGTTGCATC
Fc 3' Fw	CACCGTGTGCTCTTCCTATGCAAAC
Fc 3' Re	AAACGTTTGCATAGGAAGAGCACAC
**Human IgH**	
Sμ 5' Fw	CACCGATATTCCACCCAGGTAGTGG
Sμ 5' Re	AAACCCACTACCTGGGTGGAATATC
Sμ 3' Fw	CACCGACAGCGGGACGCAAGTAGTG
Sμ 3' Re	AAACCACTACTTGCGTCCCGCTGTC
Sγ3 3' Fw	CACCGTCGTGGATAGACAAGAACCG
Sγ3 3' Re	AAACCGGTTCTTGTCTATCCACGAC
Sγ1 3' Fw	CACCGTCGCGGACAGTTAAGAACCC
Sγ1 3' Re	AAACGGGTTCTTAACTGTCCGCGAC
Sα1 3' Fw	CACCGCTGGGTTCCTCCAGTATAG
Sα1 3' Re	AAACCTATACTGGAGGAACCCAGC

Supplementary Table 3. PCR primers used in deletion, inversion, excision circle, and surveyor assay.

Primer	Sequence (5'- 3')
**Mouse IgH (deletion, inversion, excision circle, and surveyor assay)**	
**Sμ 5' Fw**	CTTATTTCGGTTGAACATGC
**Sμ 5' Fw2**	GGACACTCAGTCAGTCAGTG
**Sμ 5' Re**	CCTGGGTCCCTCCTTACTGA
**Sμ 3' Fw**	CAGACTGTGTGCTACAGTGG
**Sμ 3' Fw2**	AGGACCTCGTTCTATAGAGG
**Sμ 3' Re**	GGTATTCATCTGAACCTTCA
**Sγ1 5' Fw**	GCCTGGTGTCAACTAGGCAG
**Sγ1 5' Re**	GGATGCCCTGGCACTGCTCT
**Sγ1 5' Re2**	CTTACCTGTAGCTACTCTGC
**Sγ1 3' Fw**	GGAAATGTCAAGAGCCAACG
**Sγ1 3' Re**	GCCTCACTTCTAGTGAACCC
**Human IgH (deletion, inversion and excision circle)**	
**Sμ 5' Fw**	CTGTAGGTCATCATCGCACC
**Sμ 5' Re**	GGTGCAACTTAGCCCAGCTC
**Sμ 3' Fw**	GAGCTGAGTGTGTGCAGCAC
**Sμ 3' Re**	CTGTGCCCTGCATGACGTCC
**Sγ3 3' Fw**	CAGTGTCCACTGGTCAAGCC
**Sγ3 3' Re**	CGTTGCAGGTGTAGGTCTGG
**Sγ1 3' Fw**	CAGTGTCCACTGGTCAAGCC
**Sγ1 3' Re**	CGTTGCAGATGTAGGTCTGG
